# An *in vivo* genome‐wide shRNA screen identifies BCL6 as a targetable biomarker of paclitaxel resistance in breast cancer

**DOI:** 10.1002/1878-0261.12964

**Published:** 2021-05-18

**Authors:** Mohammad Sultan, Jacob T. Nearing, Justin M. Brown, Thomas T. Huynh, Brianne M. Cruickshank, Emily Lamoureaux, Dejan Vidovic, Margaret L. Dahn, Wasundara Fernando, Krysta M. Coyle, Carman A. Giacomantonio, Morgan G.I. Langille, Paola Marcato

**Affiliations:** ^1^ Department of Pathology Dalhousie University Halifax NS Canada; ^2^ Department of Microbiology and Immunology Dalhousie University Halifax NS Canada; ^3^ Department of Pharmacology Dalhousie University Halifax NS Canada; ^4^ Department of Surgery Dalhousie University Halifax NS Canada

**Keywords:** breast cancer, paclitaxel, BCL6, shRNA screen, biomarkers

## Abstract

Paclitaxel is a common breast cancer drug; however, some tumors are resistant. The identification of biomarkers for paclitaxel resistance or sensitivity would enable the development of strategies to improve treatment efficacy. A genome‐wide *in vivo* shRNA screen was performed on paclitaxel‐treated mice with MDA‐MB‐231 tumors to identify genes associated with paclitaxel sensitivity or resistance. Gene expression of the top screen hits was associated with tumor response (resistance or sensitivity) among patients who received neoadjuvant chemotherapy containing paclitaxel. We focused our validation on screen hit B‐cell lymphoma 6 (BCL6), which is a therapeutic target in cancer but for which no effects on drug response have been reported. Knockdown of BCL6 resulted in increased tumor regression in mice treated with paclitaxel. Similarly, inhibiting BCL6 using a small molecule inhibitor enhanced paclitaxel treatment efficacy both *in vitro* and *in vivo* in breast cancer models. Mechanism studies revealed that reduced BCL6 enhances the efficacy of paclitaxel by inducing sustained G1/S arrest, concurrent with increased apoptosis and expression of target gene cyclin‐dependent kinase inhibitor 1A. In summary, the genome‐wide shRNA knockdown screen has identified BCL6 as a potential targetable resistance biomarker of paclitaxel response in breast cancer.

## Graphical abstract

An *in vivo* genome‐wide shRNA screen identified several potential biomarkers of paclitaxel response in breast cancer, including transcriptional repressor BCL6 as a biomarker of paclitaxel resistance. Inhibition of BCL6 in breast cancer cells and tumors resulted in increased response to paclitaxel. Paclitaxel with BCL6 inhibition resulted in increased expression of BCL6 target gene CDKN1A, sustained G1/S arrest, and increased apoptosis.

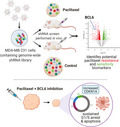

**Abbreviations**
BCL6: B‐cell lymphoma 6
BCL6i: BCL6 inhibitor
CDKN1A: cyclin‐dependent kinase inhibitor 1A
pCR: pathological complete response
RD: residual disease
TNBC: triple‐negative breast cancer

## 1 Introduction

Breast cancer remains a deadly disease for many women despite improvements in treatment and screening [1]. Treatment options include surgical resection, mastectomy, neoadjuvant or adjuvant chemotherapy, radiation, and endocrine therapy [2]. Treatment decisions are based on tumor size and grade, degree of nodal of involvement, evidence of metastasis, expression of the estrogen receptors (ER), progesterone receptors (PR), and human epidermal growth factor receptor 2 (HER2) in tumors, and the age and health of the patients. Furthermore, molecular profiling separates breast cancer into four major intrinsic subtypes: luminal A, luminal B, HER2 overexpressing, and basal‐like [3]. Each subtype is associated with an invasive/metastatic risk factor, overall prognosis, and therapeutic recommendations. In contrast to ER+, PR+, and HER2‐overexpressing breast cancers, breast tumors lacking expression of these receptors (i.e., triple‐negative breast cancers, TNBCs) are not treatable by endocrine therapies. TNBCs also tend to be more aggressive and have comparably worse outcomes [4].

In terms of outcomes, patients who achieve pathological complete response (pCR) postchemotherapy have a positive overall prognosis with lower risk of recurrence, while those who experience residual disease (RD) (non‐pCR) have poorer outcomes [5]. Identification of genes that cause resistance to chemotherapies would provide rationale for incorporating drugs that target a resistance gene, with the goal that the combination treatment would increase response and lead to improved outcomes for patients. Additionally, molecular profiling of tumors and the application of proven prognostic gene signatures can prevent under‐ and overtreatment (e.g., oncotype DX), improving outcomes and preventing exposure to unnecessary harsh treatments [6, 7].

Taxanes paclitaxel and docetaxel are commonly used chemotherapeutic drugs used in the treatment of breast [8], ovarian [9], lung [10], and pancreatic [11] cancers. Specifically, paclitaxel is used as an adjuvant chemotherapeutic agent in combination with doxorubicin in breast cancer patients with auxiliary node tumor involvement [12, 13]. Conventional paclitaxel or its albumin‐bound form is used as a second‐line therapy in breast cancer patients who have relapsed following anthracycline treatment [14, 15, 16]. Paclitaxel is also used in the treatment of breast cancer patients who overexpress HER2 in combination with the monoclonal antibody trastuzumab [17]. Recently, FDA accepted and granted priority review for the usage of oral paclitaxel in metastatic breast cancer patients [12].

Taxanes inhibit microtubule depolymerization resulting in cell cycle arrest [8]. Paclitaxel‐induced mitotic arrest can lead to DNA damage, p53 induction, and apoptosis [18, 19]. Despite the widespread use of taxanes, many patients are resistant for generally unknown reasons. In recent years, the availability of large datasets of tumor gene expression profiles combined with patient clinical data has allowed progress into generation of predictive gene signatures for taxane response [20, 21]. However, the genes identified by these methods may be biomarkers but often do not have functional relevance in chemoresistance or sensitivity, limiting their translational application into novel drug discovery for chemoresistance sensitization approaches.

In our study, we employed a genome‐wide shRNA library that had been previously used successfully to characterize the components of many pathways [22, 23]. We applied the shRNA library to MDA‐MB‐231 cells and subsequently implanted the cells in NOD/SCID mice and tumors developed. The tumor‐bearing mice were systemically treated with paclitaxel, resulting in tumor regression and the enrichment and depletion of some of the shRNAs; these shRNAs potentially target genes which mediate paclitaxel sensitivity or resistance, respectively. Top hits identified by the shRNA screen include known multidrug resistance mediator chloride channel 3 (CLCN3) [24, 25, 26], ring finger protein and ubiquitin ligase RNF144A, which lead to downregulation of DNA repair and enhanced drug response [27, 28, 29], and B‐cell lymphoma 6 (BCL6), a transcriptional repressor. Herein, we demonstrate a new role played by screen hit BCL6 in paclitaxel resistance in breast cancer and reveal associations with expression of some of the screen hits and breast cancer patient response to chemotherapy treatment that includes paclitaxel.

## 2 Materials and methods

### 2.1 Cell lines and clones

The breast cancer cell lines MDA‐MB‐231, MDA‐MB‐468, MDA‐MB‐157, MDA‐MB‐453, T47D, MCF7, BT474, SKBR3, BT20, BT549, HCC1143, HCC70, HCC1187, HCC1806, HCC1937, HS578t, and HEK293T cells were obtained from the American Type Culture Collection (ATCC, Manassas, VA, USA). SUM149 and SUM159 cells were obtained from BioIVT (previously Asterand, Westbury, NY, USA). MDA‐MB‐231, MDA‐MB‐468, MCF7, SKBR3, T47D, and HEK293T cells were grown in Dulbecco’s modified Eagle medium (DMEM; Invitrogen, Thermo Fisher Scientific, Waltham, MA, USA) supplemented with 10% FBS (Invitrogen, Thermo Fisher Scientific) and antibiotic–antimycotic (AA; Invitrogen, Thermo Fisher Scientific). MDA‐MB‐453 cells were cultured in L‐15 medium supplemented with FBS (10%) and AA; Hs578T cells were cultured in DMEM supplemented with FBS (10%), AA, and human insulin (0.01 µg·mL^−1^); and SUM149 and SUM159 cells were cultured in F‐12 Ham’s nutrient mix medium supplemented with FBS (5%), AA, 4‐(2‐Hydroxyethyl) piperazine‐1‐ethanesulfonic acid (HEPES, 1 µm; Invitrogen, Thermo Fisher Scientific), human insulin (0.01 µg·mL^−1^), and hydrocortisone (0.05 µg·mL^−1^; Invitrogen, Thermo Fisher Scientific). HCC1143, HCC1806, HCC1937, HCC1187, and HCC70 were cultured in RPMI‐1640 supplemented with FBS (10%) and AA. BT549 was cultured in RPMI‐1640 supplemented with FBS (10%), AA, human insulin (0.01 µg·mL^−1^), and glutamine (2 mm). BT20 was cultured in minimum essential medium (Invitrogen, Thermo Fisher Scientific) supplemented with FBS (10%), AA, nonessential amino acids (Invitrogen, Thermo Fisher Scientific), and sodium pyruvate. BT474 cells were grown in Iscove's modified Dulbecco's media (IMDM; Invitrogen, Thermo Fisher Scientific) supplemented with FBS (10%) and AA. Cells were cultured in a humidified 37 °C incubator with CO_2_ (5%), except for MDA‐MB‐453, which were cultured without the addition of CO_2_.

BCL6 shRNA knockdown and shRNA scramble control clones were generated using GipZ lentiviral vectors (Dharmacon, shRNA1, V2HS_271606; shRNA2, V3LHS_404721; accessed from Dalhousie University’s Faculty of Medicine Gene Analysis & Discovery Core Facility). The lentiviral supernatants were generated in HEK293T cells and applied to MDA‐MB‐231 as previously described [30]. Postselection with puromycin (1.5 µg·mL^−1^; Sigma‐Aldrich, St. Louis, MO, USA), clones were then maintained in media supplemented with puromycin (0.25 µg·mL^−1^).

### 2.2 Animal studies

Animal studies detailed in this study have been conducted in accordance with the ethical standards set by the Declaration of Helsinki and the Canadian Council on Animal Care standards and a protocol approved by Dalhousie University Committee on Laboratory Animals.

Tumor volumes were calculated for the duration of the experiments using calipers to measure the dimensions of the tumors and the volume formula (tumor volume = length × width × height/2). Once tumor/humane end points were reached in at least one mouse, experiments were terminated, and the tumor tissue was harvested and weighed.

### 2.3 *In vivo* genome‐wide shRNA screen

The Decode lentiviral shRNA library consists of the three lentiviral pools containing 10 000 shRNAs per pool (three pools, ~ 30 000 total), targeting 15 221 RefSeq mRNA accession numbers corresponding to 11 954 human genes with well‐categorized biological functions or processes were purchased from Thermo Fisher Scientific, Dharmacon (catalog # RHS5339). Following the manufacturer’s instructions, we generated three MDA‐MB‐231 shRNA pools with 100‐fold representation of the shRNA library. The genome‐wide RNAi‐transduced MDA‐MB‐231 cells were maintained as three separate pools and immediately amplified in 150 mm cell culture dishes (maintaining the minimum 100‐fold representation at all times) to achieve sufficient cell numbers to orthotopically inject 2 × 10^6^ cells per mouse into lower mammary fat pads of 8‐ to 9‐week‐old female NOD/SCID mice (Charles River, Wilmington, MA, USA, total mice 36; when the pools are later combined at the end of the experiment, this will result in 12 samples, six treatment and six no‐treatment samples). Please see Fig. S1 where this is diagramed as a flowchart. The cells were admixed 1 : 1 with high concentration phenol red‐free Matrigel (Thermo Fisher Scientific). After development of palpable tumors (day 24, 40 mm^3^ on average, calculated using the tumor volume formula, length × width × height/2), mice were divided into treatment and no‐treatment groups. Treatment mice (18 total, six of each pool) received intraperitoneal injections daily for 8 days of paclitaxel (10 mg·kg^−1^ in Cremophor EL oil, Biolyse Pharma, St. Catharines, ON, Canada); no‐treatment mice received PBS; 18 total, six of each pool). On day 32, mice were sacrificed and tumors harvested for processing.

The tumors were minced, and genomic DNA was isolated from tumors using the PureLink® DNA Purification Kit as per the manufacturer’s specifications. Molecular barcodes unique to each shRNA were then amplified from genomic DNA using Phusion Hot Start II DNA Polymerase (Thermo Fisher Scientific) and the negative selection primers included with the Decode screen (RHS5339) as per the manufacturer’s instructions. PCR products were run on a 2% agarose gel, and the resulting 250–350 bp sequence was purified with the PureLink® Gel Extraction Purification Kit (Invitrogen, Thermo Fisher Scientific) as per the manufacturer’s specifications. One microgram of gel‐purified PCR product from each pool was combined, for a total of 3 µg per sample.

The 12 samples (combined pools, 6n treatment and 6n no‐treatment controls) along with six of Custom Decode Agilent 2 × 10^5^K microarrays were sent to Ambry Genetics (Aliso Viejo, CA, USA), who labeled and hybridized the samples, scanned, and normalized the data following instruction in the Decode Array Kit. Briefly, the experimental samples were labeled with Cyanine 5‐dUTP and the reference control with Cyanine 3‐dUTP using Exo‐Klenow fragment. The labeled DNA was prepared for hybridization with Human Cot‐1 DNA and placed on the Decode array and hybridized, washed, and scanned at 5 µm resolution on an Agilent G2565CA high‐resolution scanner. Obtained data were processed through Agilent’s Feature Extraction software version 11.5.1.1 using the protocol Neg_Sel_2009 and the grid file 020719_D_F20080627_clean, and normalized gene expression values are supplied for each sample. Fold changes for each sample were calculated, and shRNAs which were overrepresented (enriched) and underrepresented (depleted) in the experimental sample (MDA‐MB‐231 with paclitaxel treatment) were identified (File S1).

### 2.4 BCL6 knockdown and BCL6i *in vivo* studies

The shRNA scramble control, BCL6 shRNA1, or BCL6 shRNA2 MDA‐MB‐231 clones or nontransduced MDA‐MB‐231 cells (2 × 10^6^ cells per mouse) were orthotopically injected into 8‐ to 9‐week‐old female NOD/SCID mice (Charles River) as described above. After development of palpable tumors (day 18–21), tumors were measured with calipers and the volumes were calculated using the tumor volume formula, length × width × height/2. The mice were then divided into treatment and no‐treatment groups. At the start of treatment, the tumors ranged from 19 to 23 mm^3^. For example, a tumor measuring 5 mm in length, 4 mm in width, and 2 mm in height equates to a tumor volume of 20 mm^3^ based on the above formula. The mice received paclitaxel (7.5 mg·kg^−1^), PBS or BCL6i (79‐6, Calbiochem; Sigma‐Aldrich, 50 mg·kg^−1^), or both paclitaxel and BCL6i daily for 7 days and then every second day for up to an additional 17 days. In these experiments, the paclitaxel dose was reduced from the initial genome‐wide screen to extend the period of treatment. The shRNA1 experiment started paclitaxel treatment on day 21 and was terminated on day 42, and the largest tumor had length of 12 mm. The shRNA2 experiment started paclitaxel treatment on day 18 and was terminated on day 35, and the largest tumor had a length of 12 mm. The BCL6i experiment started BCL6i and paclitaxel treatment on day 18 and was terminated on day 42, and the largest tumor had a length of 13 mm.

### 2.5 Quantitative PCR

RNA was extracted from cultured cells using TRIzol (Invitrogen, Thermo Fisher Scientific) combined with the PureLink RNA Kit (Invitrogen, Thermo Fisher Scientific) and incorporating a DNAse (Invitrogen, Thermo Fisher Scientific) treatment step as per the manufacturer’s protocols. Equal amounts of RNA were reverse‐transcribed with iScript™ cDNA Synthesis Kit (Bio‐Rad, Saint‐Laurent, QC, Canada), quantitative polymerase chain reaction (QPCR) was performed using SsoFast™ EvaGreen® Supermix (Bio‐Rad), and gene‐specific primers (Table S1) and efficiencies determined by standard curves were incorporated. Expression was made relative to two reference genes (pumilio RNA‐binding family member 1, PUM1, and ADP‐ribosylation factor, ARF1) and the control sample.

### 2.6 Cell proliferation assay

The day before treatment, 2 × 10^4^ MDA‐MB‐231, T47D, or MDA‐MB‐468 cells were seeded in six‐well plates and subsequently treated for 24 h with paclitaxel (7.5, 5, and 3.75 nm, as indicated in the figure legend) and/or BCL6i 79‐6 (50 µm). Viable cells were then collected and counted using Trypan Blue exclusion cell viability stain (Thermo Fisher Scientific) or continued to be cultured for another 72 h (without treatment) and then collected and counted, and viable cell numbers were calculated relative to the no‐treatment controls.

### 2.7 Cell cycle analysis

Cells were treated as described above. Cells from treatment and no‐treatment wells were collected at 24 h post‐treatment as well as 72 h post‐treatment termination and were fixed in 70% ethanol at −20 °C for 48 h. The samples were then washed with PBS and stained with 1% propidium iodide and assessed by flow cytometry using the BD FACSCanto II analyzer and then analyzed using Modfit analysis software (Verity Software House, Topsham, ME, USA).

### 2.8 Flow cytometry apoptosis analysis

Cells were treated as described above and collected for flow cytometry apoptosis analysis by staining with Alexa Fluor 647 Annexin V conjugate (Invitrogen, Thermo Fisher Scientific) and 7‐AAD (BioLegend, San Diego, CA, USA). The percentage of apoptotic/dead cells was determined by flow cytometry using a BD FACSCanto II analyzer and the FCS Express 4 Research Edition software (De Novo Software​, Pasadena, CA, USA).

### 2.9 Gene expression analysis of GEO datasets

Raw data files were obtained from the Gene Expression Omnibus (GEO) for studies GSE20194 (230 patients), GSE25055 (310 patients), and GSE25065 (198 patients). The tumor samples for all three datasets were from fine needle aspirates taken from stage I–III breast cancers before any treatment was administered. The patients from the three datasets received 6 months of neoadjuvant chemotherapy consisting of taxane, 5‐fluorouracil, cyclophosphamide, and doxorubicin. ER‐positive tumors also received endocrine therapy. Response to preoperative chemotherapy was categorized as a pCR (no residual invasive cancer in the breast or lymph nodes) or residual invasive cancer (RD). The extracted RNA samples from all three datasets were applied to HG‐U133A Affymetrix Human Genome array platforms. The raw microarray expression data from GSE20194, GSE25055, and GSE25065 were normalized using Robust Multi‐Array Average normalization within the affy R package (Fig. S2) [31]. Samples that lacked expression data or patient outcome were removed. The remaining samples were combined to create a cohort of 718‐patient tumor gene expression data (File S2). This 718‐patient tumor cohort is referred to as a cohort treated with neoadjuvant chemotherapy including paclitaxel in the Results section. The fold change in expression was calculated using the average expression for each gene, made relative to sensitive patients. The total gene expression was calculated by summing the gene expression values of the resistant genes (or summing the gene expression values of the sensitive genes) for each patient tumor and comparing the average sum of the resistant versus sensitive patient tumors (File S2). We also performed the same analysis on the cohort of patients in which we removed any patient tumors that had been treated with endocrine therapy. This reduced the number of patient tumors from 718 to 437 patients.

Similar analyses were performed for additional GEO datasets. Raw data files were obtained from GSE22513 (14 samples in duplicate, Affymetrix Human Genome U133 Plus 2.0 Array). The samples in the GSE22513 dataset are from pretreatment biopsies of stage IIA‐IIIB breast cancer. The patients in the cohort were treated with three rounds of neoadjuvant paclitaxel followed by concurrent paclitaxel and radiation. Patients defined as pCR had the absence of invasive cancer in breast and lymph nodes and non‐pCR was defined by the persistence of > 10 microscopic foci of invasive carcinoma in breast or lymph nodes. The data files were processed with the Transcriptome Analysis Console (Affymetrix, part of Thermo Fisher Scientific) to generate gene expression values, and fold change in expression was calculated as described above.

Raw data files were also obtained GSE12791 (4n, parental versus paclitaxel‐resistant MDA‐MB‐231 cells, Affymetrix HG‐U133A platform). MDA‐MB‐231 cells were treated with paclitaxel for eight cycles with each cycle including a 3‐day treatment with paclitaxel (30 nm) and followed by a 7‐day exposure to control medium. This resulted in MDA‐MB‐231 cells that were resistant to paclitaxel, and their growth was no longer inhibited by paclitaxel treatment. The data files were processed with the Transcriptome Analysis Console to generate gene expression values, and fold change in gene expression was calculated as described above.

### 2.10 Statistical analyses

All statistical analyses were performed with GraphPad Prism (GraphPad Software, San Diego, CA, USA). When multiple comparisons were made, a one‐way ANOVA followed by Tukey’s post‐test was performed. If the samples are paired (*in vitro* experiments), then the repeated measures option was utilized. Tumor growth (volumes) was modeled using simple linear regression, and the slopes of the lines were compared for differences. For patient dataset analyses, the significance in change in expression in a gene (or total gene expression) between resistant versus sensitive patients was calculated using an unpaired two‐tailed *t*‐test. For the MDA‐MB‐231 dataset analysis, the significance in change in expression in a gene between parental versus paclitaxel resistance cells was calculated using a paired *t*‐test. Significant *P* values are represented as follows: *< 0.05, **< 0.01, ***< 0.001, and ****< 0.0001.

## 3 Results

### 3.1 An *in vivo* genome‐wide shRNA screen identifies potential mediators of paclitaxel sensitivity and resistance in breast cancer

**Fig. 1:**
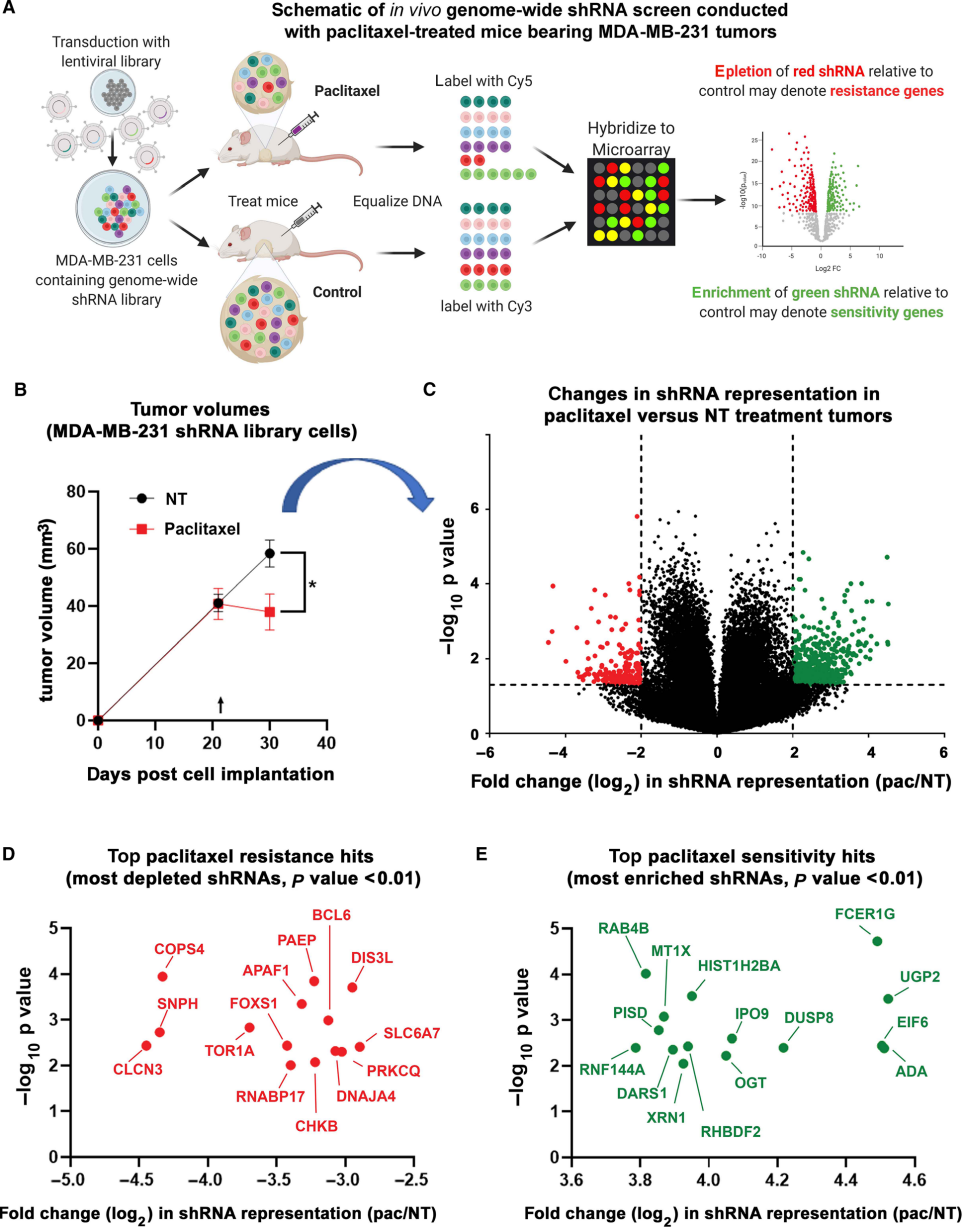
*In vivo* genome‐wide shRNA screen identifies potential mediators of paclitaxel response in breast cancer. (A) MDA‐MB‐231 cells bearing a genome‐wide shRNA library were injected into NOD/SCID mice and divided into paclitaxel treatment and control groups (*n* = 6). Following treatment termination, tumor tissues were harvested and genomic DNA extracted. The barcodes unique to each shRNA were retrieved by PCR, the amplified DNA labeled, and hybridized to microarrays to determine fold change in shRNA representation. Depleted shRNAs would theoretically be present in cells that they impart a growth disadvantage under paclitaxel treatment and thus their targets are potential resistance genes. In contrast, enriched shRNAs would theoretically be present in cells that they impart a growth advantage under paclitaxel treatment and thus their targets are potential sensitivity genes. Created with BioRender.com. (B) The average tumor volume of the NOD/SCID mice that were implanted with the MDA‐MB‐231 RNAi library cells, arrow indicates the start of intraperitoneal treatment (no‐treatment vehicle versus 10 mg·kg^−1^ paclitaxel). Error bars represent SEM. Significance was determined by performing an unpaired two‐tailed *t*‐test, and the *P* value is represented as follows: <0.05 =*. (C) The average log_2_ fold change of the individual shRNAs in the harvested tumors in the paclitaxel treatment versus no‐treatment groups was plotted against the −log_10_ *P* values (*n* = 6, unpaired *t*‐test). (D and E) The greatest depleted and enriched shRNA with *P* values < 0.01, targeting potential resistance (D) or sensitivity genes (E). [Colour figure can be viewed at wileyonlinelibrary.com]

Given the common use of paclitaxel in breast cancer treatment, we hypothesized that identifying novel effectors of paclitaxel will reveal strategies to improve treatment efficacy and improve patient outcomes. To this end, we performed an *in vivo* genome‐wide RNAi screen using the well‐characterized TNBC cell line MDA‐MB‐231 to identify novel effectors of paclitaxel response (Fig. 1A). The shRNA library MDA‐MB‐231 cells were orthotopically implanted in female NOD/SCID mice, and the mice were divided into no‐treatment and paclitaxel treatment groups. Paclitaxel treatment resulted in smaller tumors (Fig. 1B); however, cells that harbored a shRNA knockdown of a gene required for paclitaxel sensitivity would have a growth advantage. Due to the growth advantage of specific knockdowns, when the tumor‐wide DNA is analyzed from harvested tumors, there is an enrichment of the shRNA barcode sequences associated with paclitaxel sensitivity genes. In contrast, cells harboring shRNA barcode sequences that knockdown genes important for resistance to paclitaxel would have a growth disadvantage and would be depleted.

As expected, most shRNAs were not enriched or depleted in the paclitaxel‐treated tumors, with only a fraction of shRNAs being enriched or depleted greater twofold (Fig. 1C, green and red dots, File S1). We identified the most depleted and enriched shRNAs with a *P* value of < 0.01, which targeted potential paclitaxel resistance (Fig. 1D) or sensitivity (Fig. 1E) conferring genes. Encouragingly, the top resistance gene hit, CLCN3 (shRNA depleted 4.45‐fold, File S1), encodes for transporter chloride channel 3 and is a known mediator of multidrug resistance, including paclitaxel and docetaxel resistance in breast cancer cells [24, 25, 26]. Furthermore, one of the top sensitivity gene hits, ring finger protein RNF144A (shRNA enriched 3.79, File S1), encodes for an E3 ubiquitin ligase that leads to increased chemotherapy treatment response in TNBC cells by downregulating DNA repair [27, 29].

### 3.2 Some of the screen hits correlate with patient response to chemotherapy treatment including paclitaxel

**Fig. 2:**
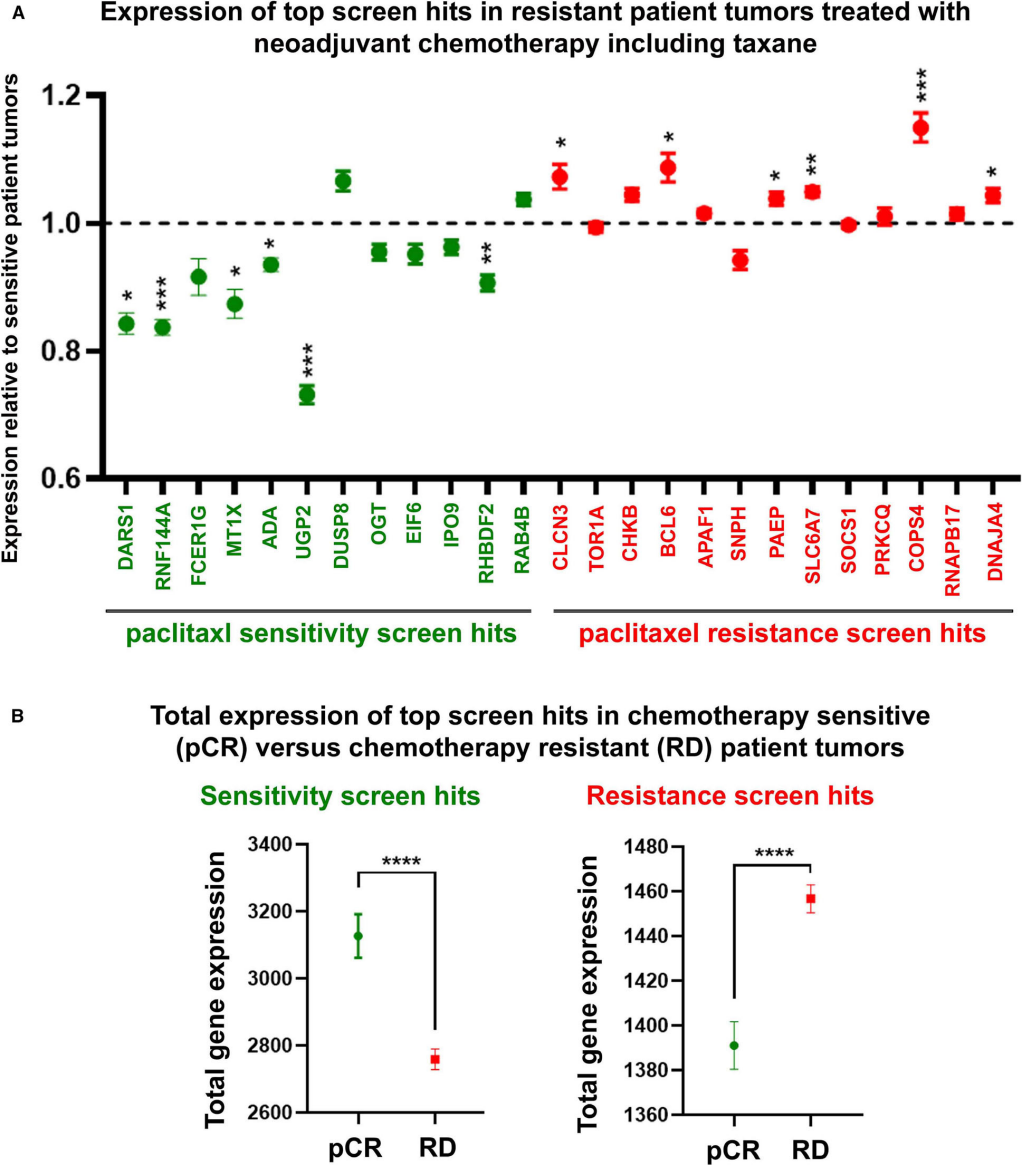
The expression of some of the screen hits is associated with response in breast cancer patients treated with chemotherapy including taxane. A cohort of patient tumor samples (*n* = 718) consisting of HG‐U133A Affymetrix Human Genome array platform and response to neoadjuvant chemotherapy that included paclitaxel was constructed using datasets GSE20194, GSE25055, and GSE25065. (A) The fold change in expression of screen hits in treatment‐resistant patient tumors (RD) was calculated relative to sensitive patient tumors (pCR). (B) Total expression of screen hits (sensitivity genes, left; resistance genes, right) was compared in sensitive patient tumors (pCR) versus resistant patient tumors (RD). Error bars represent SEM. Significance was determined by performing an unpaired two‐tailed *t*‐test comparing the expression of each gene (or the total sum of the expression of resistance genes or sensitivity genes) in the sensitive versus resistant tumors. *P* values are represented as follows: < 0.05 =*, < 0.01 =**, < 0.001 =***, and < 0.0001 =****. [Colour figure can be viewed at wileyonlinelibrary.com]

Given the wide use of taxanes in breast cancer treatment, we were able to evaluate the expression of the top screen identified genes (Fig. 1) in a cohort of patients treated with neoadjuvant chemotherapy including paclitaxel (GSE20194, GSE25055, and GSE25065, 718 patients total, File S2). Transcriptome profiling on the tumors was completed pretreatment, using the HG‐U133A microarray platform, and the patient tumors were grouped as either sensitive to treatment (pCR) or resistant (RD). Probes for expression of a few of the screen hits were missing from the datasets (or in the case of screen hit PISD, the probe was not specific to PISD) and therefore were not included in our analysis. Our analysis revealed that expression of several resistance genes, including CLCN3, BCL6, and COP9 signalosome complex subunit 4 (COPS4), was more highly expressed in the chemotherapy resistant tumors (Fig. 2A). In contrast, the expression of several sensitivity genes was lower, including top sensitivity hits UDP‐glucose pyrophosphorylase 2 (UGP2) and RNF144A, and aspartyl‐tRNA synthetase (DARS1).

We wondered if the associations were specific to triple‐negative patient tumors, given that the screen was performed in triple‐negative MDA‐MB‐231 tumors. Similar trends were observed when the analysis was repeated with the exclusion of patient tumors that had also received endocrine therapy (Fig. S3). This suggests that the associations are not specific to TNBC patient tumors. The elimination of the hormone receptor‐expressing tumors that received endocrine therapy also reduced the number of patient tumors from 718 to 437 samples. Likely, the reduction in sample number resulted in the observed reduced significance.

To assess whether expression of the screen hits together, rather than expression of individual screen hits gives more robust correlations, we calculated the total gene expression of the resistant and sensitivity screen hits in the neoadjuvant chemotherapy including paclitaxel cohort (GSE20194, GSE25055, and GSE25065, File S2). Importantly, the total gene expression of the resistance gene hits in the cohort was significantly higher in treatment‐resistant tumors (Fig. 2B, left). Similarly, the total gene expression of the sensitivity gene hits in the cohort was significantly higher in the patient tumors that achieved pCR (Fig. 2B, right). This analysis illustrates that correlations with patient tumor response are stronger when the expression of multiple gene hits is considered together instead of the expression of individual genes (as in Fig. 2A).

For comparison purposes, we performed a similar analysis with the top 14 downregulated genes and the top 13 upregulated genes in the resistant patient tumors in the same cohort. As expected, this revealed a highly significant gene expression associations (Fig. S4). Since the shRNA screen is designed to identify functional hits, it is not surprising that the screen top hits do not overlap with the top highly downregulated or upregulated genes in the patient tumor cohort. Although none of the top genes overlapped, this analysis demonstrates how some of our screen hits are appropriately down‐ or upregulated in the context of resistant patient tumors. Together, these analyses suggest that at least some of the screen hits may be useful as biomarkers or predictive tools in determining likely treatment outcome. In particular, a few resistant screen hits may be good candidates for future investigation in targeted combination therapy strategies (e.g., CLCN3, BCL6, COPS4, Fig 2A).

To further assess the potential of the screen hits, we expanded the gene expression analysis to a small patient cohort that had been treated with three rounds of neoadjuvant paclitaxel followed by concurrent paclitaxel and radiation (GSE22513, 14 samples in duplicate). In this dataset, resistant screen hits BCL6 and SNPH were significantly upregulated in the resistant patient tumors (Fig. S5). Sensitivity screen hit MTX1 was also significantly upregulated in the dataset; however, as a sensitivity hit it would be expected that the gene would be downregulated in the resistant patient tumors (i.e., hence the significant upregulation did not provide supportive evidence for this screen hit).

We also analyzed expression of the screen hits in a GEO dataset of MDA‐MB‐231 cells with acquired resistance to paclitaxel. We detected significant downregulation of sensitivity gene hits DARS1, MTX1, UGP2, OGT, and EIF6 and significant upregulation of resistance gene hits CLCN3, TOR1A, and BCL6 in the paclitaxel‐resistant cells (Fig. S6).

Together, the analyses of the datasets demonstrate the consistent upregulation of resistant screen hit BCL6 in treatment‐resistant patient tumors and paclitaxel‐resistant MDA‐MB‐231 cells (Figs 2 and S5,S6). Additionally, the availability of a small molecule inhibitor that targets resistance screen hit BCL6 (BCL6i, 79‐6) [32], and the lack of prior studies assessing the effect of BCL6 on drug response, prompted us to investigate the role of BCL6 in paclitaxel response in breast cancer.

### 3.3 Knockdown or inhibition of resistance hit BCL6 results in decreased viable cells and tumor growth in paclitaxel‐treated TNBCs

**Fig. 3:**
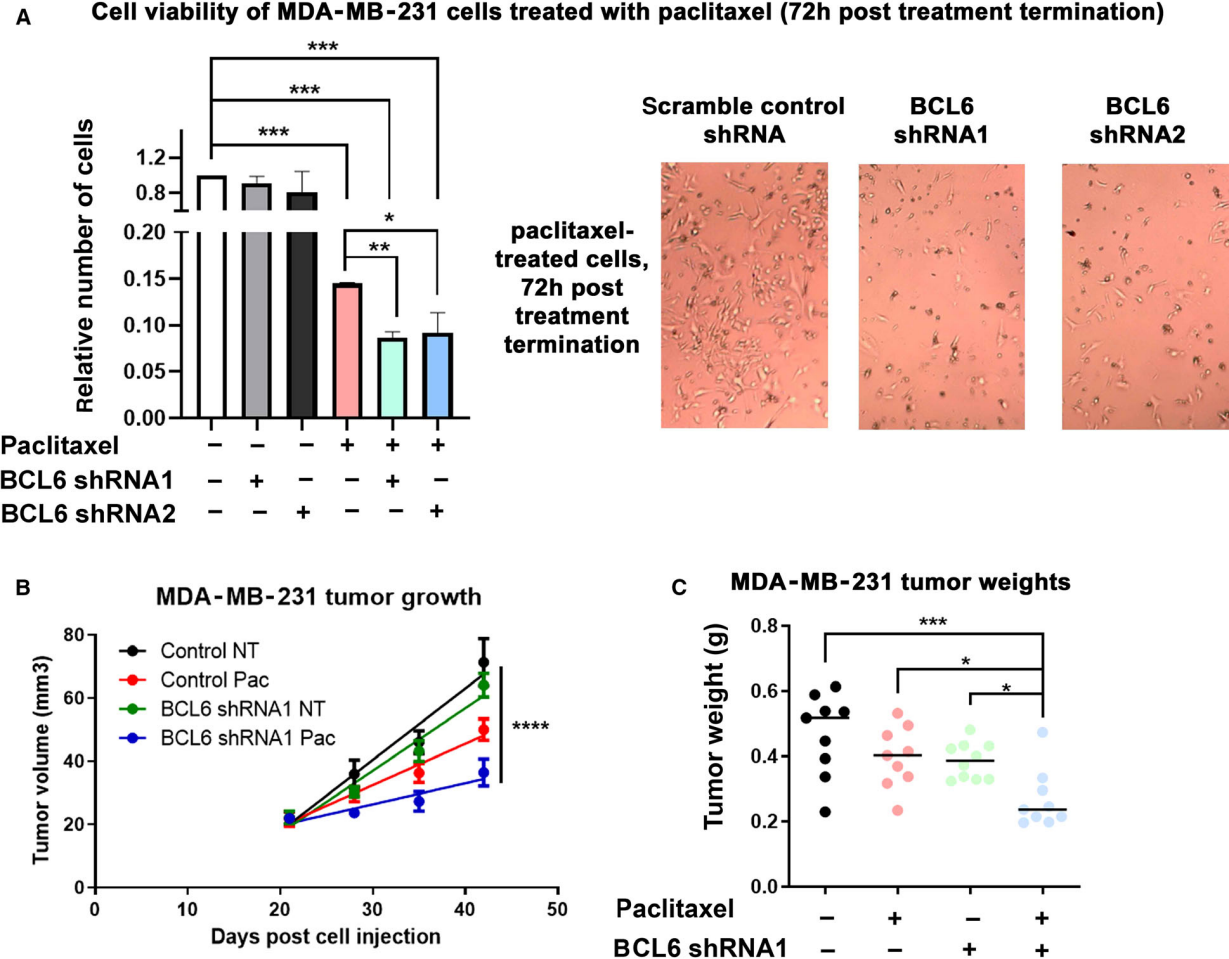
BCL6 knockdown enhances paclitaxel effect on MDA‐MB‐231 cells and tumors. (A) The effect of BCL6 knockdown (shRNA1 and 2) on *in vitro* cultured cell growth was assessed relative to a scrambled control shRNA 72 h post‐treatment termination with paclitaxel (7.5 nm) for 24 h (*n* = 4, representative microscope photographs of treated cells, right). Error bars represent standard deviation, and significance was determined by one‐way ANOVA (repeated measures) followed by Tukey’s post‐test. (B and C) The effect of BCL6 knockdown with shRNA1 and/or intraperitoneal paclitaxel treatment (started day 21 postcell injection, ended on day 42) on MDA‐MB‐231 tumors (control NT, *n* = 9; control paclitaxel, *n* = 9; BCL6 shRNA1 NT, *n* = 10; BCL6 shRNA1 paclitaxel, *n* = 9) was assessed by measuring tumor volumes (B) and final tumor weights (C). Error bars represent SEM for the panels. (B) Tumor growth was modeled using simple linear regression and the slopes of the lines compared. The slopes are significantly different from each other (*P* value = <0.0001). (C) Significance was determined by one‐way ANOVA followed by Tukey’s post‐test. (A to C) *P* values are represented as follows: < 0.05 =*, < 0.01 =**, < 0.001 =***, and < 0.0001 =****. [Colour figure can be viewed at wileyonlinelibrary.com]

We investigated the effect of BCL6 on efficacy of paclitaxel treatment by generating stable BCL6 knockdown clones in MDA‐MB‐231 cells using two different shRNAs (including the screen shRNA, designated shRNA1). Knockdown resulted in decreased BCL6 expression (Fig. S7), which did not significantly alter the proliferation of untreated cells *in vitro* (Fig. S8). *In vitro* treatment of MDA‐MB‐231 cells for 24 h with paclitaxel did not significantly reduce the number of viable cell numbers (Fig. S9). However, upon sustained cell culture for an additional 72 h post‐treatment cessation, the evidence of the 24‐h drug treatment became significant, which was pronounced in the BCL6 knockdown clones (Fig. 3A). The effects of BCL6 knockdown were more evident and significant *in vivo*. BCL6 knockdown or paclitaxel treatment modestly reduced MDA‐MB‐231 tumor growth; however, the combination of BCL6 knockdown and paclitaxel resulted in significant reduction in tumor volumes (Fig. 3B) and weights (Fig. 3C). Similar tumor growth effects were obtained with shRNA2 (Fig. S10).

**Fig. 4:**
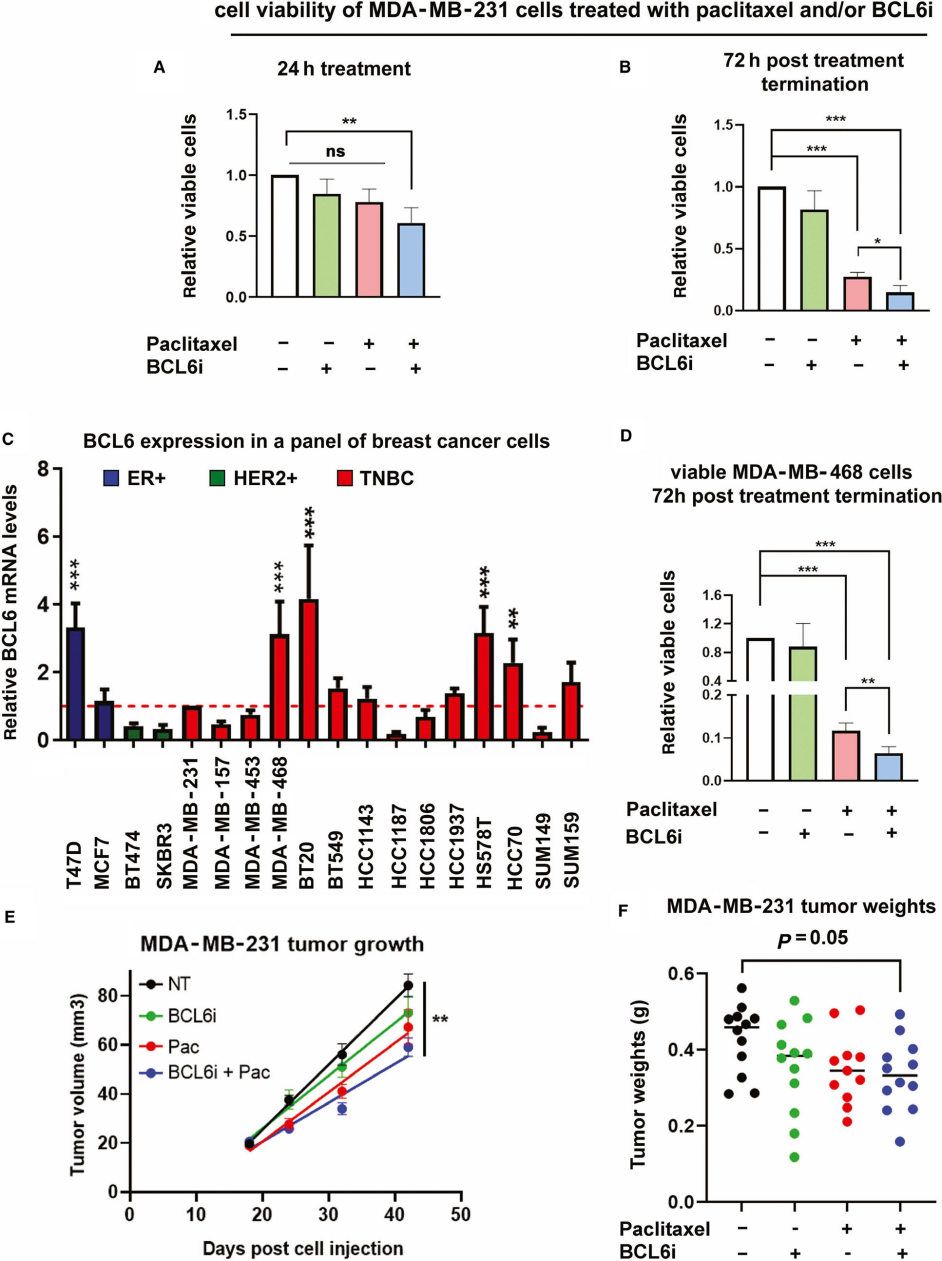
BCL6i enhances paclitaxel‐induced breast cancer cell viability and tumor regression. Trypan blue exclusion assay was used to investigate the effect of paclitaxel (7.5 nm) and/or BCL6i (50 µm) treatment on the number of viable MDA‐MB‐231 cells immediately after 24 h of treatment (A) and 72 h of post‐treatment termination (B) (*n* = 4). (C) BCL6 expression in a panel of breast cancer cell lines is assessed by qPCR (*n* = 4). (D) Trypan blue exclusion assay was used to investigate the effect of 24‐h paclitaxel (3.75 nm) and/or BCL6i (50 µm) treatment on the number of viable MDA‐MB‐468 cells 72 h of post‐treatment termination. (A to D) Error bars represent standard deviation, and significance was determined using one‐way ANOVA (repeated measures) followed by Tukey’s post‐test. (E and F) The effect of intraperitoneal BCL6i and/or paclitaxel treatment (started day 18 postcell injection and ended on day 42) on MDA‐MB‐231 tumors (*n* = 12) was assessed by measuring tumor volumes (E) and final tumor weights (F). Error bars represent standard error of the mean. (E) Tumor growth was modeled using simple linear regression and the slopes of the lines compared. The slopes are significantly different from each other (*P* value = 0.0031). (F) Significance was determined using one‐way ANOVA followed by Tukey’s post‐test. (A to F) *P* values are indicated as follows: < 0.05 =*, < 0.01 =**, and < 0.001 =***. [Colour figure can be viewed at wileyonlinelibrary.com]

We next assessed the effect of combining paclitaxel treatment with small molecule BCL6 inhibitor 79‐6 (BCL6i). This selective inhibitor of BCL6 works by inhibiting the transcriptional repression activity of BCL6. Its application in cancer cells results in increased expression of target genes (e.g., CDKN1A, p21) and decreased cell proliferation [32]. *In vitro* treatment of MDA‐MB‐231 cells for 24 h with 7.5 nm paclitaxel and/or 50 µm BCL6i resulted in a decrease in viable cells, that was only significant in the combination treatment (Fig. 4A). Upon sustained cell culture for a further 72 h post‐treatment cessation, there was a significant decrease in viable cells with either treatment alone, which was more pronounced (additive) with the combination treatment (Fig. 4B).

To expand these studies to additional cell lines, we assessed expression of BCL6 in a panel of breast cancer cell lines to identify a cell line which had high levels of BCL6 (Fig. 4C). BCL6 expression was higher in several TNBCs cell lines (e.g., MDA‐MB‐468 cells) as well as ER+ T47D cells. We selected MDA‐MB‐468 and T47D cells for analysis. We treated the cells for 24 h with paclitaxel and/or the BCL6i. The treatment did not have a significant effect in the number of viable MDA‐MB‐468 and T47D cells after 24 h of treatment (Fig. S11,S12). However, 72 h post‐treatment termination, we observed the delayed effects of the drugs on cell viability, which resulted in a more pronounced reduction in viable MDA‐MB‐468 and T47D cell numbers in the combination treatment (Figs 4D and S12). We did not note any overt differences in terms of the sensitivity of the three cell lines to BCL6 inhibition in the context of paclitaxel treatment. Possibly, the threefold differences of BCL6 (transcript levels, Fig. 4C) in three cell lines (MDA‐MB‐231, MDA‐MB‐468, and T47D cells) were insufficient to result in discernable differences in terms of sensitivity to BCL6 inhibition.

We assessed the effects of the drugs *in vivo*. Mice bearing palpable MDA‐MB‐231 tumors were treated with paclitaxel, the BCL6i, or the combination of both drugs. The combination treatment resulted in more pronounced decrease in tumor volumes and weights (Fig. 4E,F). Together, these data (Figs 3 and 4) confirmed the screen result in identifying BCL6 as a potential paclitaxel resistance mediator (Fig. 1), which upon knockdown or inhibition results in a further cancer cell/tumor growth disadvantage in the context of paclitaxel treatment.

### 3.4 BCL6 knockdown promotes sustained G1/S phase cell cycle arrest and apoptosis in paclitaxel‐treated TNBC cells

**Fig. 5:**
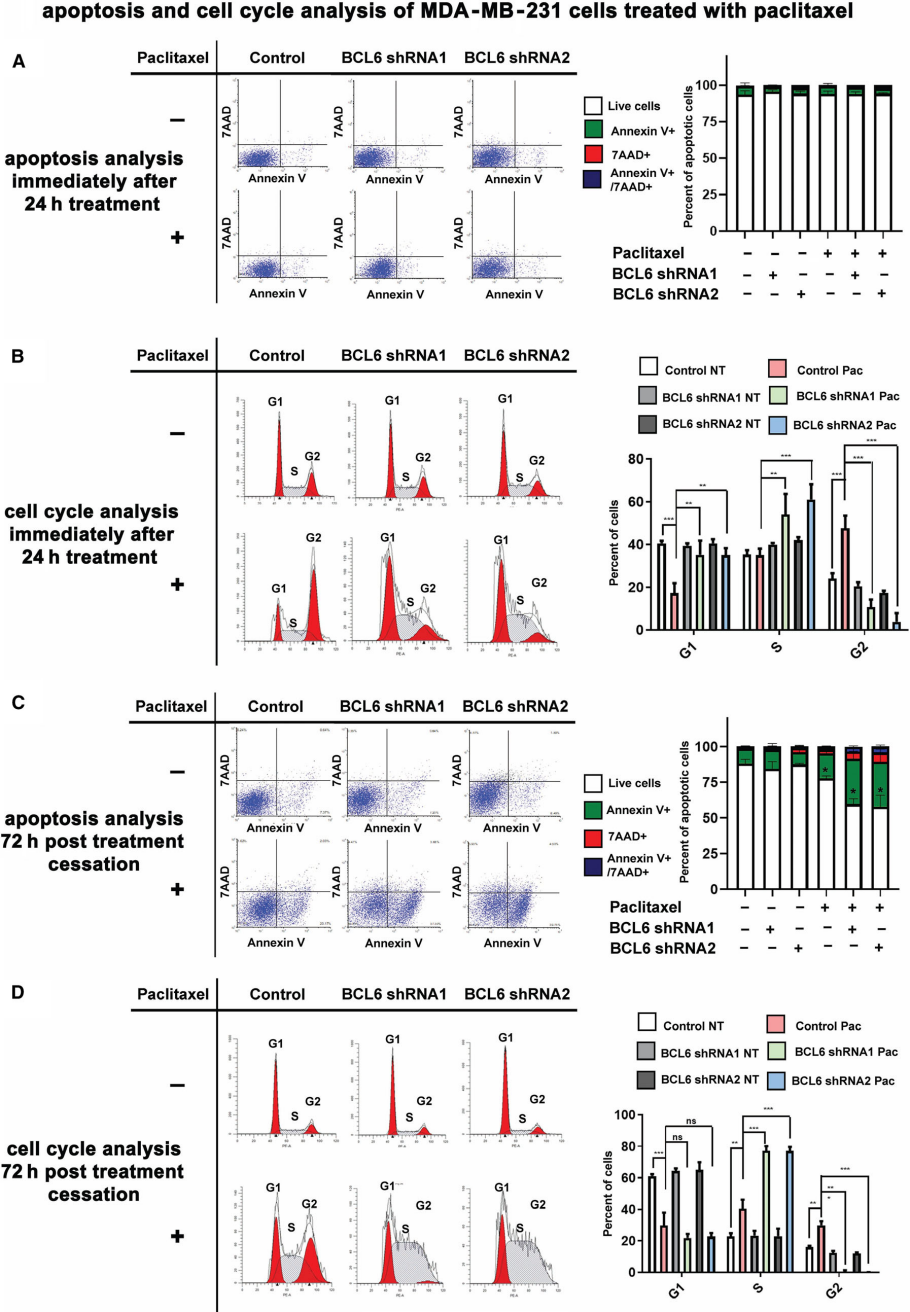
BCL6 knockdown induces a sustained G1/S phase cell cycle arrest in MDA‐MB‐231 cells treated with paclitaxel. MDA‐MB‐231 BCL6 shRNA1 and 2 or scramble shRNA control cells were treated for 24 h with paclitaxel (7.5 nm) and analyzed for apoptotic/dead cells immediately after by flow cytometry analysis of Annexin V+/7‐AAD+ cells (A) or cell cycle by propidium staining of fixed cells (B), *n* = 4. Representative dot plots and cell cycle analyses are depicted. Alternatively, the cells were cultured for an additional 72 h post‐treatment termination and then assessed for apoptotic/dead cells (C) or cell cycle (D), *n* = 4. A to D) Error bars represent standard deviation, and significance was determined by one‐way ANOVA (repeated measures) followed by Tukey’s post‐test. *P* values are indicated as follows: < 0.05 =*, < 0.01 =**, and < 0.001 =***. [Colour figure can be viewed at wileyonlinelibrary.com]

To determine the mechanism of how decreased BCL6 leads to enhanced paclitaxel effects, we assessed the effects of BCL6 knockdown and paclitaxel treatment on cell cycle arrest and apoptosis, both of which are known to be induced by paclitaxel. Following 24 h of paclitaxel treatment, we performed flow cytometry apoptosis analysis via Annexin V and 7‐AAD staining of unfixed cells and cell cycle analysis via PI staining of fixed cells. This revealed no changes in the level of apoptotic cells in any of the treatment or untreated groups at 24 h post‐treatment (Fig. 5A). This was expected since there was not a significant change in the number of viable cells after 24 h of treatment (Fig. S9). In contrast, the cell cycle analysis revealed significant changes in the cell cycle progression of BCL6 knockdown cells following 24 h of paclitaxel treatment, that were distinct from the paclitaxel‐treated shRNA scramble control cells (Fig. 5B). As previously reported [33], paclitaxel induced a G2/M cell cycle arrest in the MDA‐MB‐231 shRNA scramble control cells; however, we noted a shift toward G1/S phase of the cell cycle in the paclitaxel‐treated BCL6 knockdown cells.

To determine whether the change in cell cycle progression at 24 h would translate to increased apoptosis in the days following treatment and explain the delayed effects on the reduced number of viable cells (Fig. 3A), we repeated the cell apoptosis and cell cycle analyses at this later time point. This revealed a significant increase in the number in apoptotic cells in the BCL6 knockdown cells treated with paclitaxel (Fig. 5C), in agreement with the cell viability assay (Fig. 3A). Cell cycle analysis of samples collected 72 h post‐treatment termination showed the scramble control shRNA paclitaxel‐treated cells were still partly in G2/M cell cycle arrest (Fig. 5D), however, to a lesser extent than observed immediately after the 24‐h treatment (Fig. 4B). In contrast, the BCL6 knockdown cells remained arrested in G1/S phases, with almost no cells in the G2 phase (Fig. 5D). These results suggest that the 24‐h paclitaxel treatment leads to a sustained G1/S arrest in BCL6 knockdown, which is still in effect 72 h post‐termination of treatment, leading to the observed delayed effects on reduced cell numbers and increased apoptosis (Figs 3A and 5C). Together, these findings are consistent with reduced BCL6 levels enhancing paclitaxel activity in the breast cancer cells by promoting a more sustained G1/S phase cell cycle arrest instead of the more transitory G2/M phase cell cycle arrest observed in the shRNA scramble control cells.

### 3.5 BCL6 knockdown and inhibition increases cyclin‐dependent kinase inhibitor 1A expression in paclitaxel‐treated TNBC cells

**Fig. 6:**
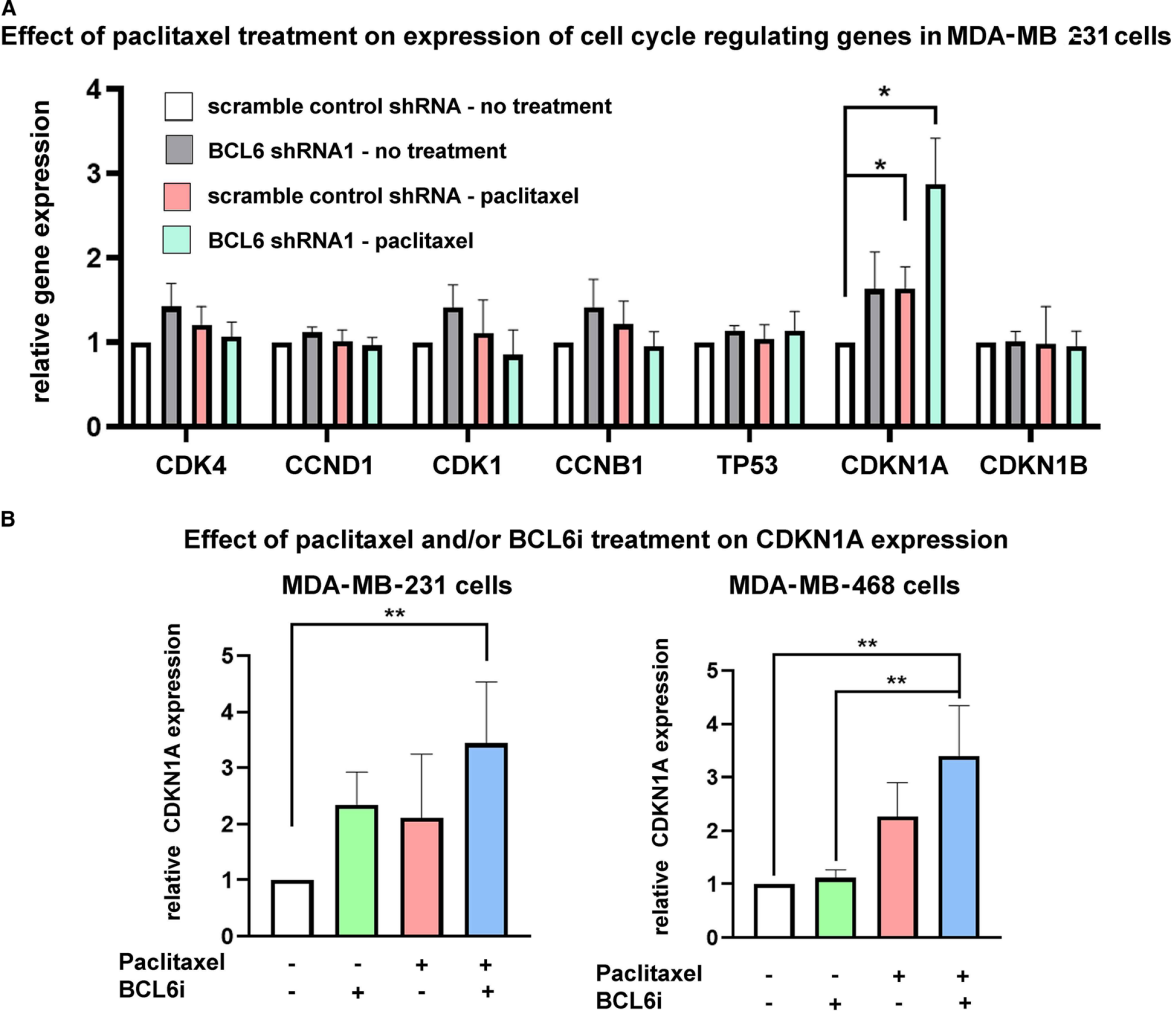
Silencing or inhibiting BCL6 in the context of paclitaxel treatment is associated with increased expression of CDKN1A in TNBC cells. (A) QPCR analysis of expression of cell cycle regulator genes in MDA‐MB‐231 BCL6 shRNA1 cells relative to scramble control shRNA cells following 24 h of paclitaxel treatment (7.5 nm, *n* = 4). (B) MDA‐MB‐231 cells (left) or MDA‐MB‐468 (right) cells treated with paclitaxel and/or BCL6i (50 µm) for 24 h were analyzed for CDKN1A expression by qPCR (*n* = 4). Error bars represent standard deviation, and significance was determined using one‐way ANOVA (repeated measures) followed by Tukey’s post‐test; *P* value < 0.05 =* and < 0.01 =**. [Colour figure can be viewed at wileyonlinelibrary.com]

Previous reports have demonstrated the important role of BCL6 as a transcriptional repressor involved in silencing genes with function in cell cycle progression and apoptotic pathways [34, 35]. Thus, we hypothesized that the effect of BCL6 knockdown on cell cycle arrest induced by paclitaxel treatment (Fig. 5) could be due to the altered gene expression of cell cycle regulators. While several check points exist in the mammalian cell cycle, we focused on the regulators of cell cycle progression from G1 to the S phase and from the G2 to the M phase, since these were the phases affected in our assays by paclitaxel treatment (Fig. 5). These include gene coding for cyclins, cyclin‐dependent kinases, and major regulators of cell cycle regulation pathways such as p53 and cyclin‐dependent kinase inhibitor 1A and 1B (CDKN1A) and (CDKN2A). QPCR revealed that the expression of many of these cell cycle regulators was unaffected by BCL6 knockdown in MDA‐MB‐231 cells (shRNA1) treated with 7.5 nm paclitaxel for 24 h; the exception was CDKN1A which was upregulated (Fig. 6A). We confirmed that CDKN1A gene expression was increased when BCL6 was knocked down by shRNA2 in MDA‐MB‐231 cells treated with paclitaxel (Fig. S13). Additionally, BCL6i treatment resulted in an increase in CDKN1A expression in paclitaxel‐treated MDA‐MB‐231 and MDA‐MB‐468 cells (Fig. 6B). These results are in accordance with a previous study that demonstrates that BCL6 inhibits CDKN1A expression and cell cycle arrest in B‐cell lymphoma [34]. CDNK1A encodes p21, which induces G1/S arrest [36, 37], and is a likely explanation for the G1/S cell cycle arrest observed in BCL6 knockdown cells treated with paclitaxel and BCL6i+paclitaxel‐treated cells. Together, these findings suggest that the benefit of inhibiting BCL6 in combination with paclitaxel in breast cancer could be at least in part explained by the release of CDKN1A suppression.

## 4 Discussion

The common use of taxanes in cancer treatment in general and in breast cancer specifically [8] has led to the investigation of resistance mechanisms and methods to overcome this resistance to increase treatment efficacy. Some studies focused on specific mechanisms, such as the upregulation of the multidrug resistance pumps [38], β tubulin overexpression [39], and downregulation of apoptotic pathways [40]. Other studies employed *in vitro* genome‐wide screening technologies to identify novel mediators of paclitaxel resistance [41, 42]; however, *in vitro* screens may not reveal factors which depend upon the tumor microenvironment. This sparked our interest in preforming a genome‐wide shRNA screen *in vivo* to identify genes which play a role in paclitaxel response.

Our screen identified several genes which may promote paclitaxel resistance and sensitivity in breast cancer tumors. These genes include top resistance screen hit CLCN3, which has been well characterized as a mediator of multidrug resistance (e.g., cisplatin, etoposide, and both taxanes) [24, 25, 26], and sensitivity screen hit RNF144A, which downregulates DNA repair in the context of etoposide‐induced DNA damage [27, 29, 43]. Two potential mechanisms are reported for CLCN3‐mediated drug resistance: upregulation of key multidrug resistance efflux pump p‐glycoprotein and vesicular acidification resulting in increased drug sequestration [24, 26]. In contrast, RNF144A expression in TNBCs contributed to etoposide‐induced DNA damage and apoptosis by ubiquitination of DNA‐dependent protein kinase catalytic subunit (DNA‐PKcs), inhibiting the DNA repair pathway [29]. In cells with persistent and/or severe DNA damage, RNF144A is proposed to mediate p53‐induced apoptosis through downregulation of DNA‐PKcs [27]. Paclitaxel similarly results in p53‐mediated apoptosis via DNA damage that occurs from prolonged cell cycle arrest [18, 19]. Therefore, RNF144A could contribute to increased paclitaxel sensitivity by halting repair to DNA damage resulting from the taxane treatment.

With the mechanisms of increased drug resistance and sensitivity of screen hits CLCN3 and RNF144A already described, we focused our investigation on another resistance screen hit, BCL6. The transcriptional repressor has been investigated in breast cancer [44, 45], but not in the context of drug and/or taxane response. The availability of BCL6‐specific inhibitors, associations with breast cancer patient tumor response to chemotherapy treatment including paclitaxel, and increased expression of BCL6 in MDA‐MB‐231 cells with acquired paclitaxel resistance provided further rationale for studying the role of BCL6 in paclitaxel response in breast cancer.

BCL6 has been primarily studied as a mediator of tumor progression in B‐cell lymphoma [46]. Our data suggest that decreased BCL6 increases paclitaxel response in breast cancer by shifting the cell cycle arrest induced by paclitaxel toward a more prolonged G1/S arrest. This was associated with an increase in apoptosis and CDKN1A expression, which is consistent with a previous report that BCL6 is transcriptional repressor of the cell cycle arrest gene [34].

The effect of BCL6 inhibition on cell viability was observed in both TNBC and ER+ paclitaxel‐treated cells, suggesting that inhibition of BCL6 could be incorporated as a potential strategy across breast cancer subtypes and not just in a subset of patients. However, it is important to note that while BCL6 inhibition did increase the response of paclitaxel, the effect was modest in the tumor study. This suggests that targeting BCL6 either needs to be optimized in terms of treatment timing and dose (e.g., continued daily administration instead of every 2 days), or that it is insufficient to target BCL6 alone and make a major impact in increasing response to paclitaxel treatment. It may be that a cocktail of inhibitors targeting a few resistance mediators is required. It is possible that some of these other potential targets were identified in our screen.

The validation of screen hits BCL6 in this study, and CLCN3 [24, 25, 26] and RNF144A [27, 29, 43] by others, would imply that at least some of the other screen hits are also likely valid. Of interest for future study are top sensitivity hit UGP2 and resistance hit COPS4 which were the most lowly expressed and highly expressed screen hits in the large chemotherapy treatment‐resistant breast cancer patient tumor cohort. Although not studied in the context of breast cancer, UGP2 expression (both high and low) has been implicated in the progression of other cancers [47, 48], and our data suggest a potential uncharacterized role for UGP2 in breast cancer in the context of treatment. COPS4 is part of the multisubunit COP9 that plays roles in DNA repair and cell cycle regulation in part through modulating the activity of E3 ubiquitin ligases [49]. Although not yet studied in the context of drug response, knockdown of COPS4 in MDA‐MB‐231 cells was recently shown to increase apoptosis [49]. This is reminiscent of some of our findings with resistance screen hit BCL6.

## 5 Conclusions

The success of the oncotype DX as a predictive tool for treatment response in ER+ positive breast cancer patients [50] suggests that genes signature could be useful for predicting taxane response. Several taxane‐associated gene signatures have been developed by analyzing the transcriptome of patient tumors prior to treatment, leading to the identification of predictive gene signatures [21, 51]. To this end, we analyzed the expression of the screen identified response mediators in relation to treatment response in patient tumor cohorts. The data suggest that there may be predictive value in at least a few gene hits and further investigation along these lines is warranted. In conclusion, this study demonstrates the potential of genetic screens performed *in vivo* to identify mediators and/or biomarkers of chemotherapy response. In our study, this approach has revealed the possible strategy of overcoming chemotherapy resistance in breast cancer patients by incorporating BCL6i.

## Conflict of interest

The authors declare no conflict of interest.

## Author contributions

MS conceived and designed the study, involved in collection and/or assembly of data, analyzed and interpreted the data, wrote the manuscript, and approved the final version of the manuscript. JTN, JMB, TTH, BMC, EL, DV, MLD, WF, and KMC involved in collection and/or assembly of data, analyzed and interpreted the data, and approved the final version of the manuscript. MGIL and CAG analyzed and interpreted the data and approved the final version of the manuscript. PM conceived and designed the study, analyzed and interpreted the data, wrote the manuscript, and approved the final version of the manuscript.

### Peer Review

The peer review history for this article is available at https://publons.com/publon/10.1002/1878‐0261.12964.

## Supporting information


**Fig. S1**. Flowchart of the shRNA screen showing mouse numbers and shRNA pools.
**Fig. S2**. Normalized expression plots of GSE20194, GSE25055 and GSE25065.
**Fig. S3**. Removal of the endocrine therapy treated patients results in fewer of the screen hits being significantly associated with response in breast cancer patients treated with chemotherapy including taxane.
**Fig. S4**. Expression of the top downregulated and upregulated genes in chemotherapy (including taxane) resistant patient tumors relative to sensitive patient tumors.
**Fig. S5**. Increased expression of resistance screen hits BCL6 and SNPH is associated with treatment resistance in breast cancer patients treated with neoadjuvant paclitaxel followed by concurrent paclitaxel and radiation.
**Fig. S6**. The expression of some sensitivity screen genes is downregulated, and expression of some resistance genes is upregulated in paclitaxel‐resistant MDA‐MB‐231 cells.
**Fig. S7**. BCL6 knockdown in MDA‐MB‐231 cells.
**Fig. S8**. BCL6 knockdown does not alter the number of viable MDA‐MB‐231 cells cultured over 9 days.
**Fig. S9**. BCL6 knockdown in combination with paclitaxel does not significantly reduce the number of viable MDA‐MB‐231 cells after 24 h of treatment.
**Fig. S10**. BCL6 knockdown with shRNA2 enhances paclitaxel‐induced regression of MDA‐MB‐231 tumors.
**Fig. S11**. Treatment with BCL6i or paclitaxel treatment (alone or in combination) do not significantly reduce the number of viable MDA‐MB‐468 cells after 24 h of treatment.
**Fig. S12**. Paclitaxel and BCL6i combination treatment reduce the number of viable T47D cells 72 h post‐treatment termination.
**Fig. S13**. BCL6 knockdown with shRNA2 in the context of paclitaxel treatment is associated with increased expression of CDKN1A in MDA‐MB‐231 cells.
**Table S1**. Primer sequences and efficiencies used in QPCR.

**File S1**. The shRNA screen data in an excel file.

**File S2**. The patient cohort expression data in an excel file.

## Data Availability

The data that support the findings of this study are openly available in the Gene Expression Omnibus, https://www.ncbi.nlm.nih.gov/geo/, GSE20194, GSE25055, GSE25065, GSE22513, and GSE12791. The results published here are in part based upon data generated by the TCGA Research Network: https://www.cancer.gov/tcga.
