# Supplementary material for: An in vivo genome‐wide shRNA screen identifies BCL6 as a targetable biomarker of paclitaxel resistance in breast cancer
Source: Mol Oncol. 2021 May 18;15(8):2046–64. doi: 10.1002/1878-0261.12964 (PMC8333778; doi:10.1002/1878-0261.12964)
Supplement: Supplementary file 1 — Fig. S1. Flowchart of the shRNA screen showing mouse numbers and shRNA pools. Fig. S2. Normalized expression plots of GSE20194, GSE25055 and GSE25065. Fig. S3. Removal of the endocrine therapy treated patients results in fewer of the screen hits being significantly associated with response in breast cancer patients treated with chemotherapy including taxane. Fig. S4. Expression of the top downregulated and upregulated genes in chemotherapy (including taxane) resistant patient tumors relative to sensitive patient tumors. Fig. S5. Increased expression of resistance screen hits BCL6 and SNPH is associated with treatment resistance in breast cancer patients treated with neoadjuvant paclitaxel followed by concurrent paclitaxel and radiation. Fig. S6. The expression of some sensitivity screen genes is downregulated, and expression of some resistance genes is upregulated in paclitaxel‐resistant MDA‐MB‐231 cells. Fig. S7. BCL6 knockdown in MDA‐MB‐231 cells. Fig. S8. BCL6 knockdown does not alter the number of viable MDA‐MB‐231 cells cultured over 9 days. Fig. S9. BCL6 knockdown in combination with paclitaxel does not significantly reduce the number of viable MDA‐MB‐231 cells after 24 h of treatment. Fig. S10. BCL6 knockdown with shRNA2 enhances paclitaxel‐induced regression of MDA‐MB‐231 tumors. Fig. S11. Treatment with BCL6i or paclitaxel treatment (alone or in combination) do not significantly reduce the number of viable MDA‐MB‐468 cells after 24 h of treatment. Fig. S12. Paclitaxel and BCL6i combination treatment reduce the number of viable T47D cells 72 h post‐treatment termination. Fig. S13. BCL6 knockdown with shRNA2 in the context of paclitaxel treatment is associated with increased expression of CDKN1A in MDA‐MB‐231 cells. Table S1. Primer sequences and efficiencies used in QPCR. [file MOL2-15-2046-s003.pdf]

## SUPPLEMENTARY FIGURES and TABLES

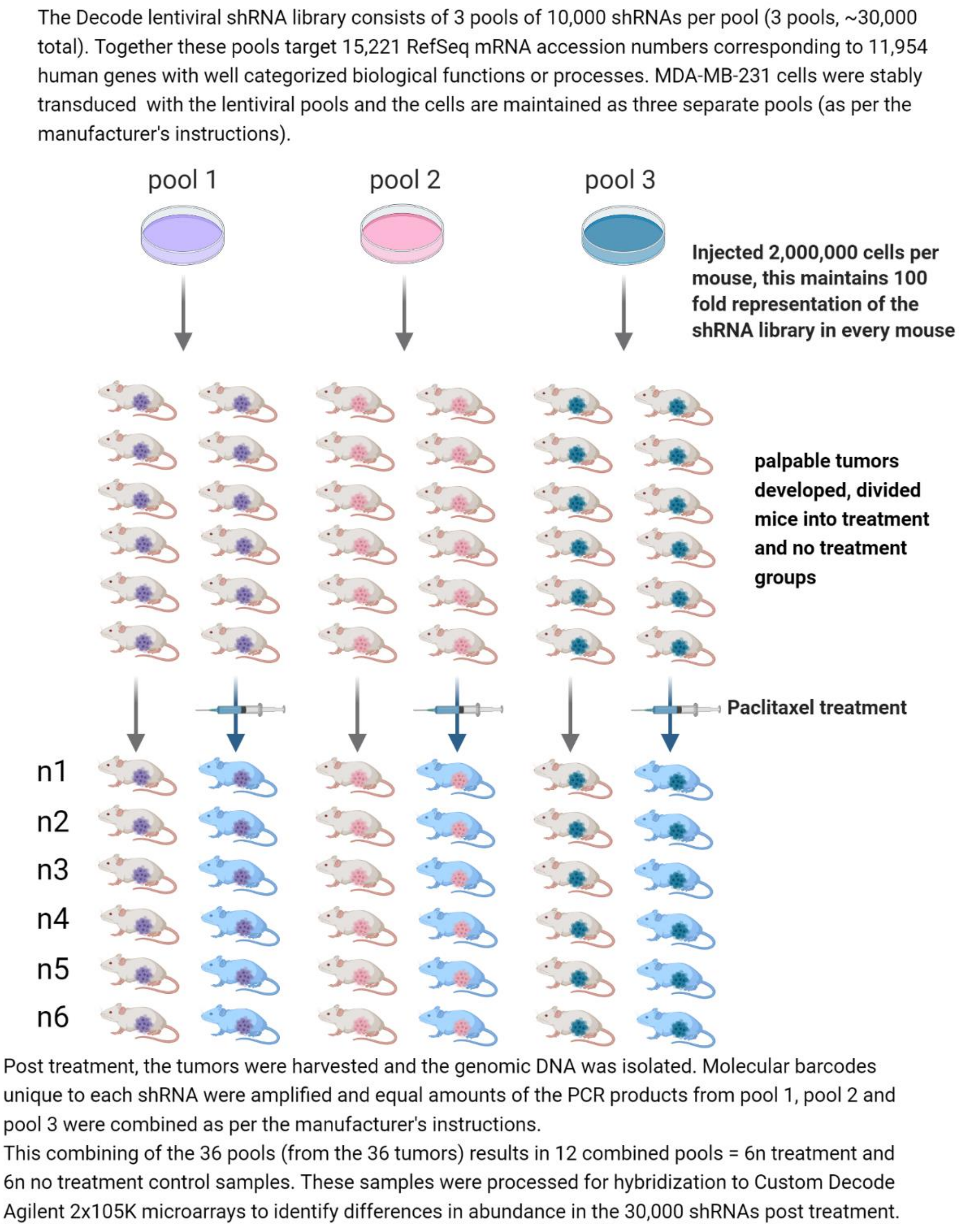


**Figure S1. Flowchart of the shRNA screen showing mouse numbers and shRNA pools.** MDA-MB-231 cells were transduced with 30,000 different shRNAs (3 pools of 10,000 shRNA lentiviral vectors) and maintained as three separate pools. To obtain an experiment with 6n, 36 mice were implanted with MDA-MB-231 shRNA library cells (pool 1, 2 or 3) resulting in 6 treatment and 6 no treatment control samples when the pools are combined for final analysis (post tumor harvesting). The figure was created with BioRender.com.

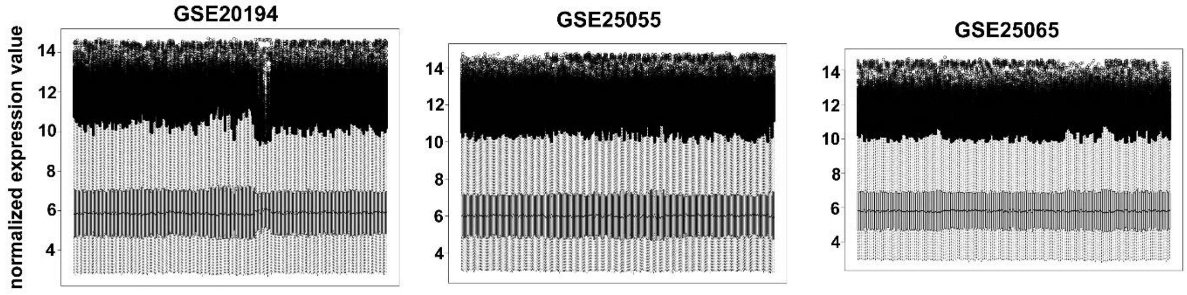


**Figure S2. Normalized expression plots of GSE20194, GSE25055 and GSE25065.** The raw microarray expression data was then normalized using Robust Multi-Array Average normalization within the affy R package.

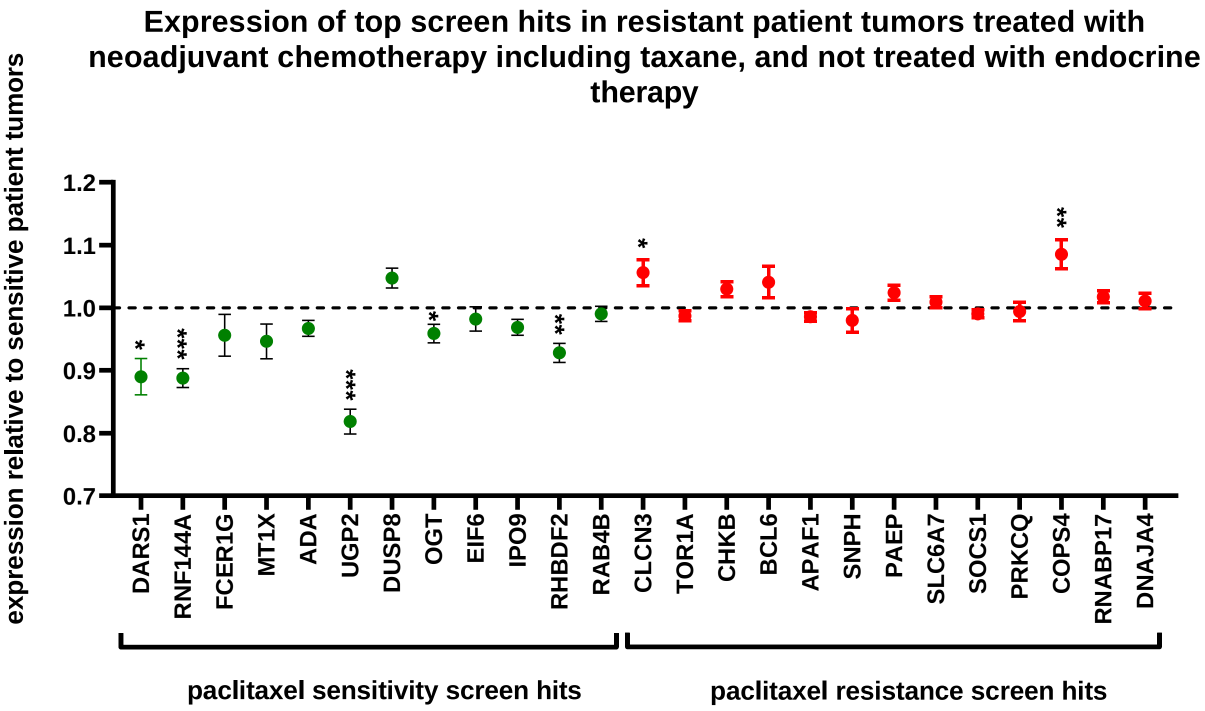


**Figure S3. Removal of the endocrine therapy treated patients results in fewer of the screen hits being significantly associated with response in breast cancer patients treated with chemotherapy including taxane.** The cohort of patient tumor samples from Figure 2 was reanalyzed with the exclusion of patients that had received endocrine treatment (i.e., hormone receptor positive tumors). This reduced the sample number to 437 patient tumors from the 718 tumors analyzed in Figure 2. The fold change in expression of screen hits in treatment resistant patient tumors (residual disease), was calculated relative to sensitive patient tumors (pathological complete response, pCR). Significance was determined by performing an unpaired t-test. P values are represented as follows: <0.05 =*, < 0.01 =**, <0.001 =***. Error bars represent SEM.

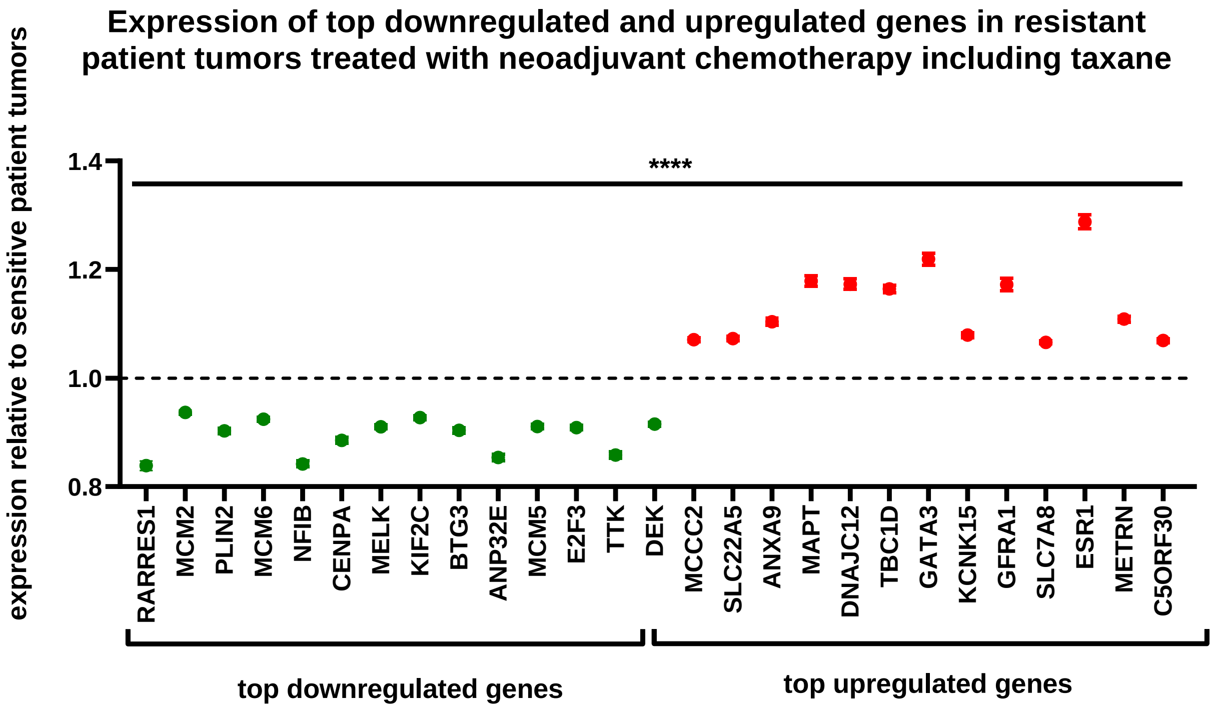


**Figure S4. Expression of the top downregulated and upregulated genes in chemotherapy (including taxane) resistant patient tumors relative to sensitive patient tumors.** The cohort of patient tumor samples from Figure 2 was reanalyzed to identify the top down and upregulated genes in the resistant patient tumors (relative to the sensitive tumors). Significance was determined by performing an unpaired t-test comparing the expression of each gene in the sensitive versus resistant tumors. The p values were extremely significant for all the genes (<0.0001 =****). Error bars represent SEM.

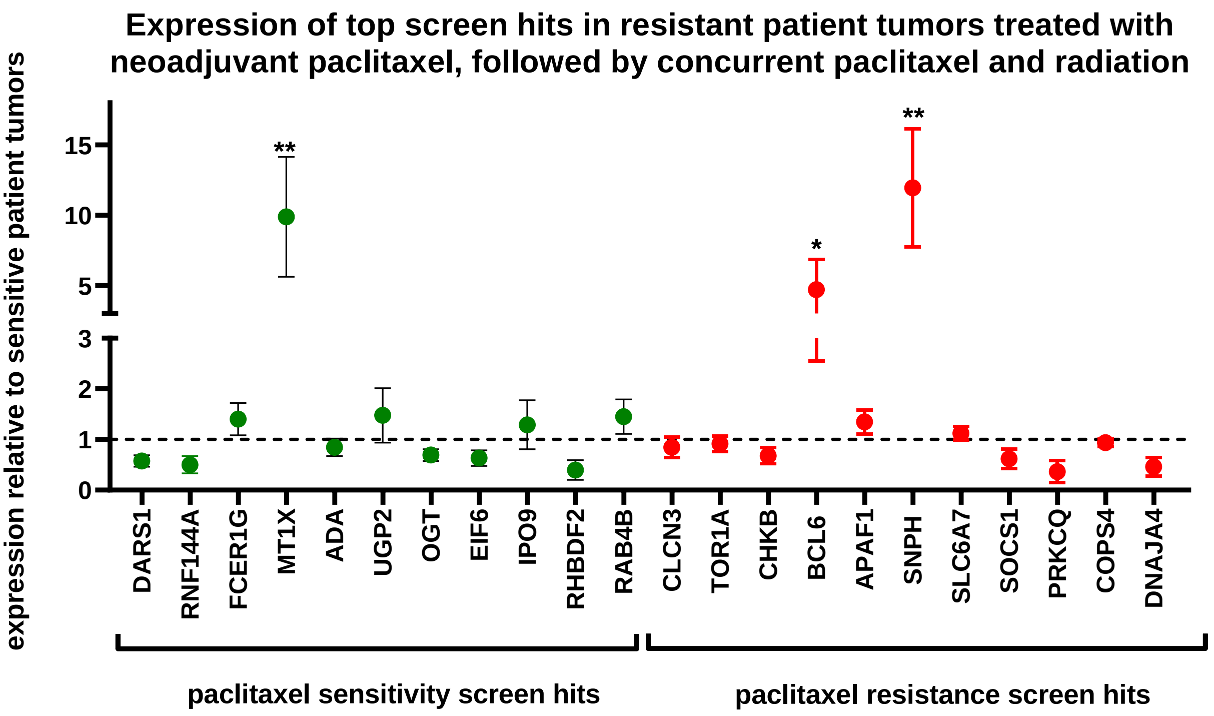


**Figure S5. Increased expression of resistance screen hits BCL6 and SNPH is associated with treatment resistance in breast cancer patients treated with neoadjuvant paclitaxel followed by concurrent paclitaxel and radiation.** Patients with stage IIA-IIIB breast cancer were treated with three cycles of paclitaxel followed by concurrent paclitaxel/radiation. Patients with pCR were defined by the absence of invasive cancer in breast and lymph nodes and non-pCR was defined by the persistence of >10 microscopic foci of invasive carcinoma in breast or lymph nodes. GSE22513 contains the gene expression profiling data (HG-U133A Affymetrix Human Genome array platform) generated from 14 pre-treatment biopsy samples in duplicate. The fold change in expression of screen hits in treatment resistant patient tumors (non-pCR), was calculated relative to sensitive patient tumors (pCR). Significance was determined by performing an unpaired t-test comparing the expression of each gene in the sensitive versus resistant tumors. Significant p values are represented as follows: <0.05 =*, <0.01 =**.

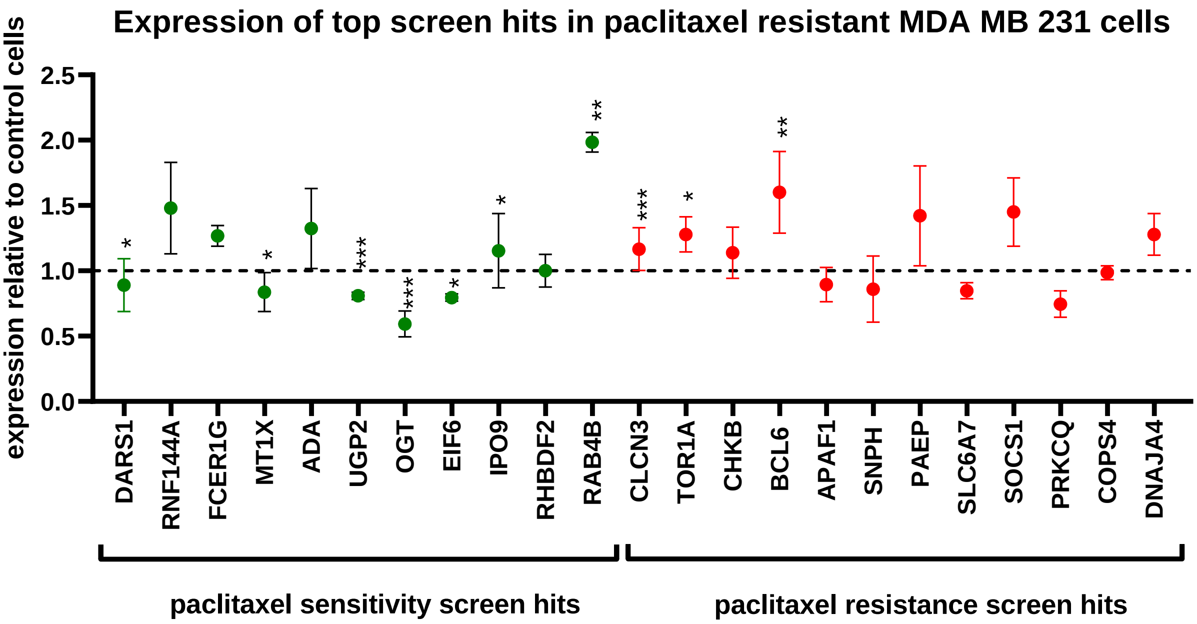


**Figure S6. The expression of some sensitivity screen genes is downregulated, and expression of some resistance genes is upregulated in paclitaxel resistant MDA MB 231 cells.** The expression of the screen hits was extracted from GSE12791 (HG-U133A Affymetrix Human Genome array platform, n=4). MDA MB 231 cells were repeatedly treated with paclitaxel (30nM) for 8 cycles. The surviving MDA MB 231 cells were resistant to paclitaxel and their growth was no longer inhibited by paclitaxel treatment. The paclitaxel resistant cells were compared to parental MDA MB 231 cells. Significance was determined by performing a paired t-test comparing the expression of each gene in the parental versus paclitaxel resistant cells. Significant p values are represented as follows: <0.05 =*, <0.01 =**, <0.001 =***.

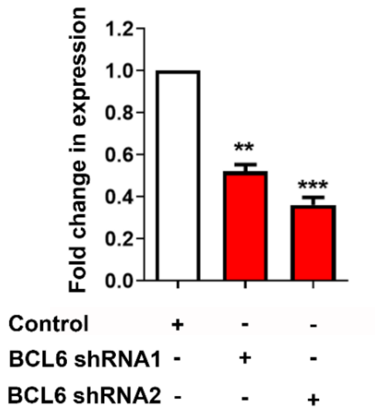


**Figure S7. BCL6 knockdown in MDA MB 231 cells.** BCL6 knockdown with two different shRNAs was confirmed using QPCR (shRNA1= screen shRNA, n=3). Error bars represent standard deviation. Significance was determined using repeated measures one-way ANOVA (repeated measures, Tukey's post test). Significant p values are represented as follows: < 0.01 =**, <0.001 =***.

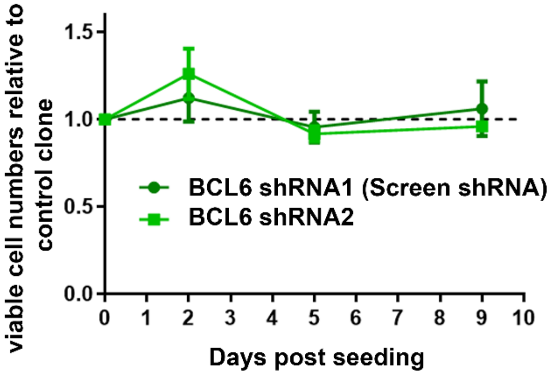


**Figure S8. BCL6 knockdown does not alter the number of viable MDA MB 231 cells cultured over 9 days.** Equal numbers of scramble control or BCL6 shRNA clones were seeded and the number of viable cells quantified over 9 days. This was determined by counting the live cells (Trypan Blue exclusion staining) and made relative to the shRNA scramble clone (n = 3). Error bars represent standard deviation. Significance was tested using repeated measures one-way ANOVA followed by Tukey's post test, and no condition was significantly different.

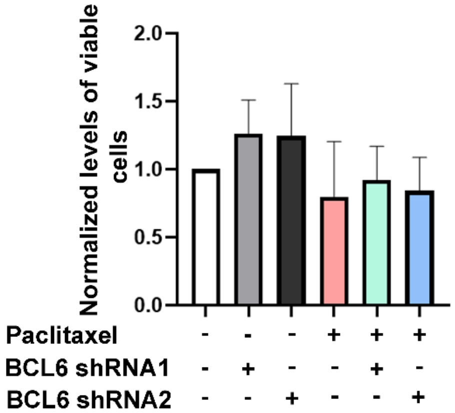


**Figure S9. BCL6 knockdown in combination with paclitaxel does not significantly reduce the number of viable MDA-MB-231 cells after 24 hours of treatment.** Trypan blue cell viability assay was preformed on control and BCL6 knockdown clones following 24h of paclitaxel treatment and the number of viable cells was reported relative to untreated control (n=3). Significance was tested using repeated measures one-way ANOVA followed by Tukey's post test, and no condition was significantly different.

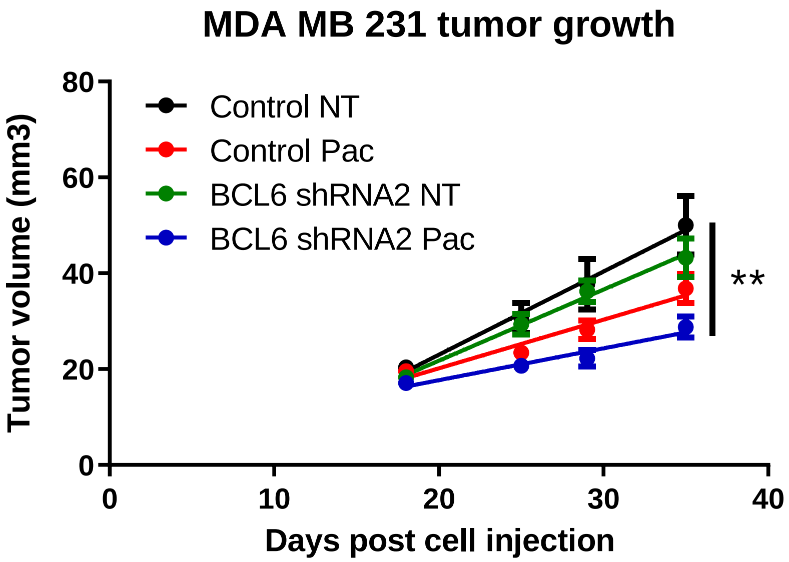


**Figure S10. BCL6 knockdown with shRNA2 enhances paclitaxel induced regression of MDA MB 231 tumors.** BCL6 role as a potential resistance mediator was assessed in NOD/SCID mice using BCL6 shRNA2 by comparing tumor volumes. Error bars represent SEM. Tumor growth was modeled using simple linear regression and the slopes of the lines compared. The slopes are significantly different from each other (p value = 0.0022, represented as **). Paclitaxel treatment started on day 18 and ended on day 35 (termination of experiment).

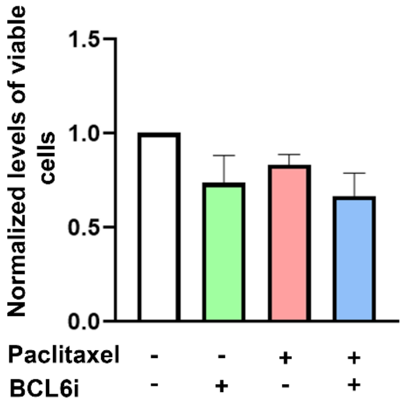


**Figure S11. Treatment with BCL6i or paclitaxel treatment (alone or in combination) do not significantly reduce the number of viable MDA MB 468 cells after 24 hours of treatment.** Trypan Blue cell viability assay was preformed on MDA MB 468 cells following 24h of paclitaxel (3.75nM) and/or BCL6i treatment (50µM) and the level of viable cells was reported relative to untreated control (n=3). Significance was determined using one-way ANOVA (repeated measures) followed by Tukey's post test, no condition was significantly different.

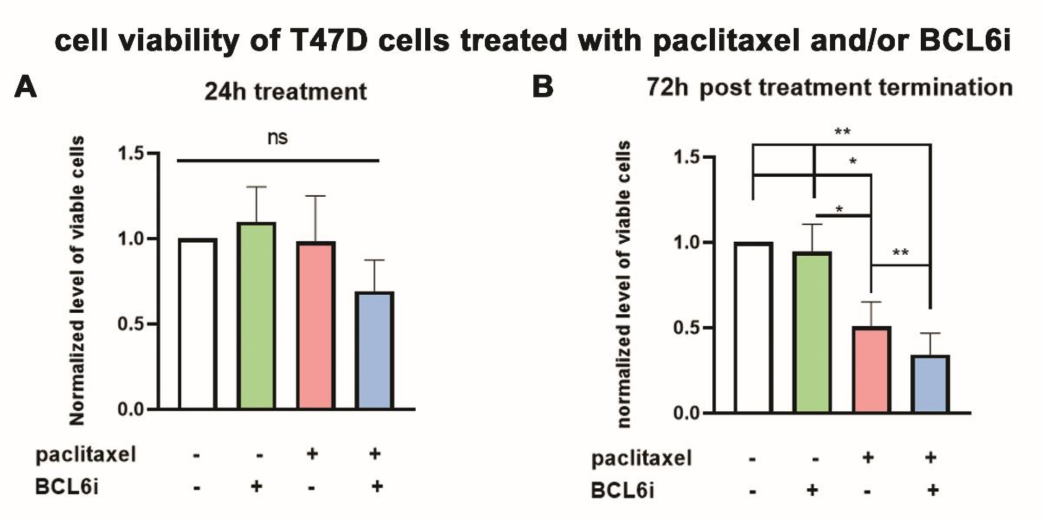


**Figure S12. Paclitaxel and BCL6i combination treatment reduce the number of viable T47D cells 72 hours post treatment termination.** Trypan Blue cell viability assay was preformed on T47D cells following 24h of paclitaxel (5nM) and/or BCL6i (50µM) treatment **(A)**, or 72h post treatment termination **(B)**. The level of viable cells was reported relative to untreated control (n=4). Significance was determined using one-way ANOVA (repeated measures) followed by Tukey's post test. Significant p values are represented as follows: < 0.05 =*, < 0.01 =**.

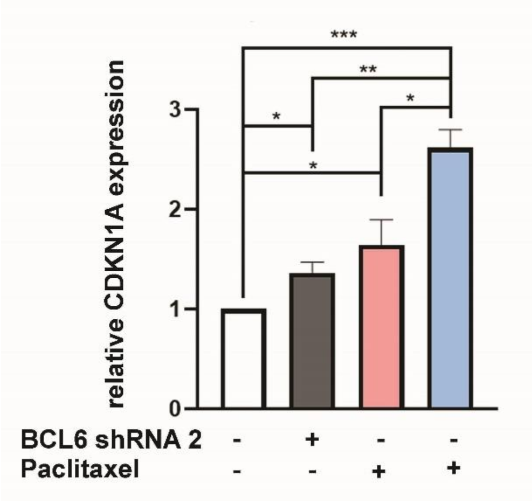


**Figure S13. BCL6 knockdown with shRNA2 in the context of paclitaxel treatment is associated with increased expression of CDKN1A in MDA MB 231 cells**. QPCR analysis of CDKN1A mRNA levels in MDA MB 231 cells in BCL6 shRNA2 cells versus scramble control shRNA cells following 24h of paclitaxel treatment (7.5nM, n=4). Error bars represent standard deviation and significance was determined using one-way ANOVA (repeated measures) followed by Tukey's post test, p value <0.05 =*, <0.01 =**, <0.001 =***.

| Primer | Direction | Sequence | Efficiency |
|---|---|---|---|
| CDKN1B | Forward | CCCCTAGAGGGCAAGTACGA | 89.7 |
| | Reverse | GCGGGGGTCTGTAGTAGAAC | |
| TP53 | Forward | CAGATCCGTGGGCGTGAG | 101.7 |
| | Reverse | CTGGGCATCCTTGAGTTCCAA | |
| BCL6 | Forward | GCCTCCTCGTGAAGAGTTCC | 93.7 |
| | Reverse | TTGTTCTCCACCACCTCACG | |
| CDKN1A (P21) | Forward | GCGACTGTGATGCGCTAATG | 94.5 |
| | Reverse | GAAGGTAGAGCTTGGGCAGG | |
| ARF1 | Forward | GTGTTCGCCAACAAGCAGG | 103.8 |
| | Reverse | CAGTTCCTGTGGCGTAGTGA | |
| PUM1 | Forward | GGCGTTAGCATGGTGGAGTA | 109.1 |
| | Reverse | CATCCCTTGGGCCAAATCCT | |
| CDK4 | Forward | GGAAACTCTGAAGCCGACCA | 115.9 |
| | Reverse | GCAGGGATACATCTCGAGGC | |
| CCND1 | Forward | TGAGGAGCCCCAACAACTTC | 98.3 |
| | Reverse | CCGGGTCACACTTGATCACT | |
| CDK1 | Forward | GGAAGGGGTTCCTAGTACTGC | 104.7 |
| | Reverse | TCCTGCATAAGCACATCCTGA | |
| CCNB1 | Forward | AGGCGAAGATCAACATGGCA | 107.8 |
| | Reverse | AGCTGTTCTTGGCCTCAGTC | |

**Table S1. Primer sequences and efficiencies used in QPCR.**
